# Genomic analysis and identification of a novel superantigen, SargEY, in *Staphylococcus argenteus* isolated from atopic dermatitis lesions

**DOI:** 10.1128/msphere.00505-24

**Published:** 2024-07-11

**Authors:** Fatkhanuddin Aziz, Junzo Hisatsune, Hisaya K. Ono, Junko Kajimura, Liansheng Yu, Kanako Masuda, Hiroki Kitagawa, Yusuke Sato'o, Koji Yahara, Mika Yamaoka, Akio Nakane, Hiroshi Kawasaki, Shoko Obata, Ayano Fukushima-Nomura, Yoshihiro Ito, Meiji Soe Aung, Masayuki Amagai, Siti Isrina Oktavia Salasia, Hiroki Ohge, Yoichiro Kusunoki, Motoyuki Sugai

**Affiliations:** 1 Department of Bacteriology, Graduate School of Biomedical and Health Sciences, Hiroshima City, Hiroshima, Japan; 2 Veterinary Technology Program, Department of Bioresources Technology and Veterinary, Vocational College, Universitas Gadjah Mada, Yogyakarta, Indonesia; 3 Antimicrobial Resistance Research Center, National Institute of Infectious Diseases, Higashimurayama, Tokyo, Japan; 4 Laboratory of Zoonoses, Kitasato University School of Veterinary Medicine, Towada, Aomori, Japan; 5 Department of Molecular Biosciences, Radiation Effects Research Foundation, Hiroshima City, Hiroshima, Japan; 6 Department of Infectious Diseases, Hiroshima University Hospital, Hiroshima City, Hiroshima, Japan; 7 Division of Bacteriology, Department of Infection and Immunity, School of Medicine, Jichi Medical University, Shimotsuke-shi, Tochigi, Japan; 8 Department of Microbiology and Immunology, Hirosaki University, Hirosaki, Aomori, Japan; 9 Department of Dermatology, Keio University School of Medicine, Shijuku-ku, Tokyo, Japan; 10 Laboratory for Skin Homeostasis, RIKEN Center for Integrative Medical Sciences, Yokohama, Kanagawa, Japan; 11 Laboratory for Developmental Genetics, RIKEN, Center for Integrative Medical Sciences, Yokohama, Kanagawa, Japan; 12 Department of Hygiene, Sapporo Medical University School of Medicine, Sapporo, Hokkaido, Japan; 13 Department of Clinical Pathology, Faculty of Veterinary Medicine, Universitas Gadjah Mada, Yogyakarta, Indonesia; University of Napoli Federico II, Naples, Italy

**Keywords:** *Staphylococcus*, *argenteus*, superantigen, enterotoxins, human T-cell, TCR Vα

## Abstract

During surveillance of *Staphylococcus aureus* in lesions from patients with atopic dermatitis (AD), we isolated *Staphylococcus argenteus*, a species registered in 2011 as a new member of the genus *Staphylococcus* and previously considered a lineage of *S. aureus*. Genome sequence comparisons between *S. argenteus* isolates and representative *S. aureus* clinical isolates from various origins revealed that the *S. argenteus* genome from AD patients closely resembles that of *S. aureus* causing skin infections. We previously reported that 17%–22% of *S. aureus* isolated from skin infections produce staphylococcal enterotoxin Y (SEY), which predominantly induces T-cell proliferation via the T-cell receptor (TCR) Vα pathway. Complete genome sequencing of *S. argenteus* isolates revealed a gene encoding a protein similar to superantigen SEY, designated as SargEY, on its chromosome. Population structure analysis of *S. argenteus* revealed that these isolates are ST2250 lineage, which was the only lineage positive for the SEY-like gene among *S. argenteus*. Recombinant SargEY demonstrated immunological cross-reactivity with anti-SEY serum. SargEY could induce proliferation of human CD4^+^ and CD8^+^ T cells, as well as production of TNF-α and IFN-γ. SargEY showed emetic activity in a marmoset monkey model. S_arg_EY and SET (a phylogenetically close but uncharacterized SE) revealed their dependency on TCR Vα in inducing human T-cell proliferation. Additionally, TCR sequencing revealed other previously undescribed Vα repertoires induced by SEH. S_arg_EY and SEY may play roles in exacerbating the respective toxin-producing strains in AD.

**IMPORTANCE:**

*Staphylococcus aureus* is frequently isolated from active lesions of atopic dermatitis (AD) patients. We reported that 17%–22% of *S. aureus* isolated from AD patients produced a novel superantigen staphylococcal enterotoxin Y (SEY). Unlike many *S*. *aureus* superantigens that activate T cells via T-cell receptor (TCR) Vß, SEY activates T cells via TCR Vα and stimulates cytokine secretion. *Staphylococcus argenteus* was isolated from AD patients during the surveillance for *S. aureus*. Phylogenetic comparison of the genome indicated that the isolate was very similar to *S. aureus* causing skin infections. The isolate encoded a SEY-like protein, designated SargEY, which, like SEY, activated T cells via the TCR Vα. ST2250 is the only lineage positive for SargEY gene. ST2250 *S. argenteus* harboring a superantigen SargEY gene may be a novel staphylococcal clone that infects human skin and is involved in the exacerbation of AD.

## INTRODUCTION

*Staphylococcus argenteus* is a Gram-positive bacterium belonging to the family Staphylococcaceae, previously categorized within the *Staphylococcus aureus* lineage. Initially perceived as less resistant to antimicrobials and lacking in virulence factor genes, *S. argenteus* was considered less pathogenic than *S. aureus* (1–3). However, recent observations indicate a rising incidence of human infections, animal contamination, and foodborne cases involving *S. argenteus* (4, 5). Notably, reports increasingly link *S. argenteus* to virulence factors associated with *S. aureus*, particularly superantigen-related genes (6). Many of these genes have been identified through PCR amplification using primers specific to *S. aureus* genes, suggesting a high degree of nucleotide sequence similarity between the two species.

In humans, *S. argenteus* has been isolated from various clinical conditions, including bacteremia (7, 8), wound infections (9), bone and joint infections (10), sepsis (11), and atopic dermatitis (AD) (12). Our previous research indicated that 13% of *S. aureus* isolated from AD patients harbored the gene for staphylococcal enterotoxin Y (SEY) (13). SEY comprises three variants (subtypes 1–3), originating from three distinct clonal complexes (CC121, CC20, and CC59). Notably, SEY uniquely stimulates specific T-cell subsets via the T-cell receptor (TCR) Vα, in contrast to most *S. aureus* superantigens, which are known for their TCR Vβ specificity. Staphylococcal superantigens transcribed by *S. aureus* are recognized as crucial virulence factors implicated in various diseases, including lethal sepsis, infective endocarditis, and kidney infections (14, 15). Upon stimulation by superantigens, T cells undergo vigorous proliferation and release abnormally high levels of proinflammatory cytokines and chemokines, leading to clinical manifestations such as fever, hypotension, toxic shock, and other illnesses (16–18). Therefore, elucidating the superantigens identified in *S. argenteus* is crucial for understanding their precise bioactivity and role in disease progression.

In this study, we characterized *S. argenteus* isolated from active lesions of patients with atopic dermatitis and analyzed a gene encoding a highly homologous exotoxin with SEY, as transcribed in *S. aureus*. We demonstrated that this SEY-like toxin can induce T-cell proliferation and cytokine production in human peripheral blood mononuclear cells (PBMCs). Furthermore, we explored other biological characteristics of *S. argenteus* and compared them to their *S. aureus* counterparts.

## RESULTS

### Identification of a SEY-like gene in *S. argenteus* strains

**Fig 1:**
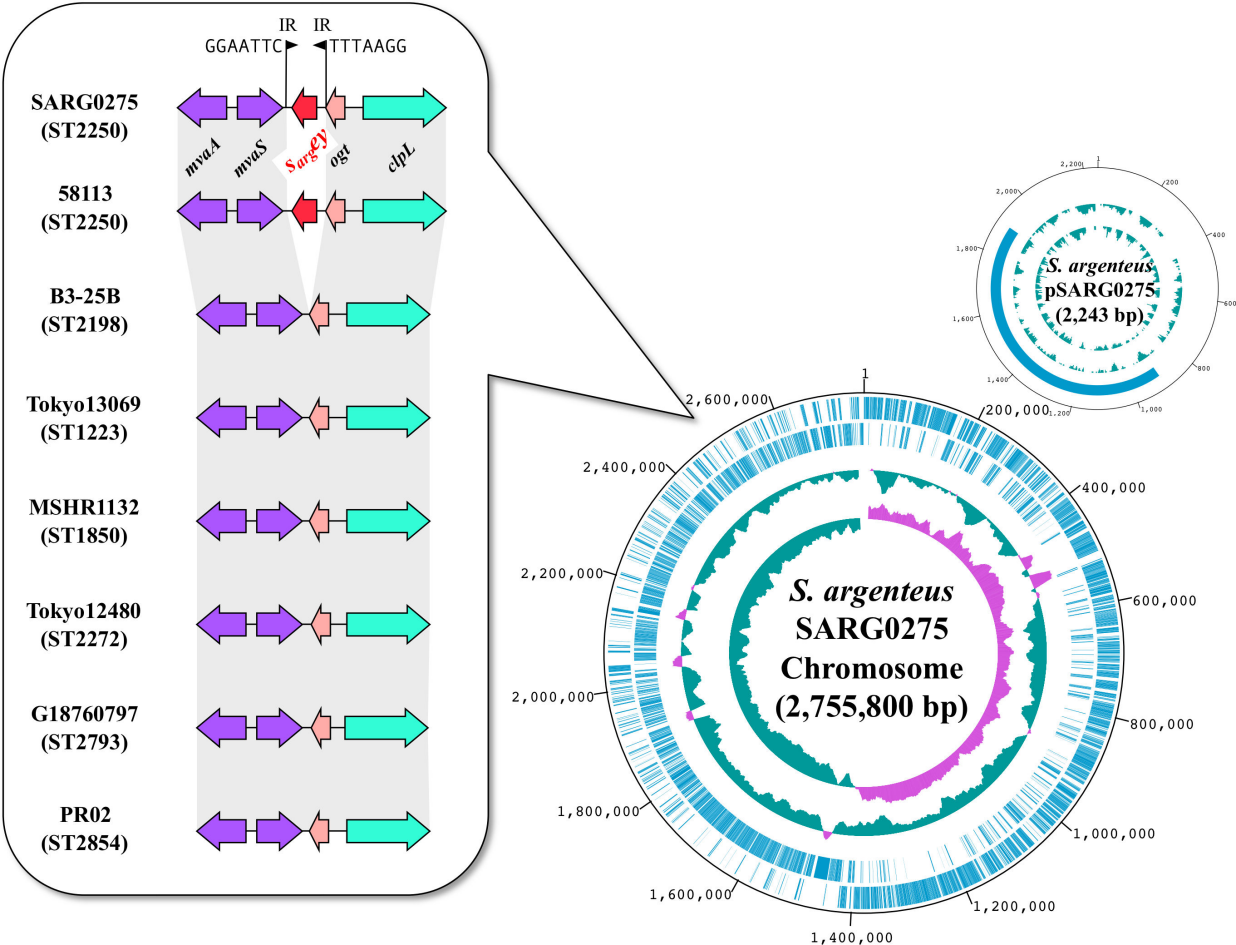
Complete Genome Sequence of ST2250 *s*_(_*_arg_*_)_*ey*-positive *S. argenteus* SARG0275. Circular representation of the *S. argenteus* chromosome and plasmid. Beginning with the outer region, the circles show nucleotide positions in base parts, predicted CDSs transcribed on the forward (clockwise) and reverse (counterclockwise) DNA strands, and percentage GC content (purple and green, respectively, represent the regions with higher and lower GC contents compared to the average value for the entire genome), and the GC skew curve. The position of *s*_(_*_arg_*_)_*ey* on the chromosome is indicated by balloon shapes. Black up-pointing triangles indicate the inverted repeat. Arrows indicate coding sequences (CDS); *mvaA* gene encoding 3-hydroxy-3-methylglutaryl CoA reductase, *mvaS* gene encoding 3-hydroxy-3-methylglutaryl CoA synthase, *ogt* gene encoding methylated-DNA-[protein]-cysteine S-methyltransferase, *clpL* gene encoding ATP-dependent Clp protease ATP-binding subunit ClpL.

**Fig 2:**
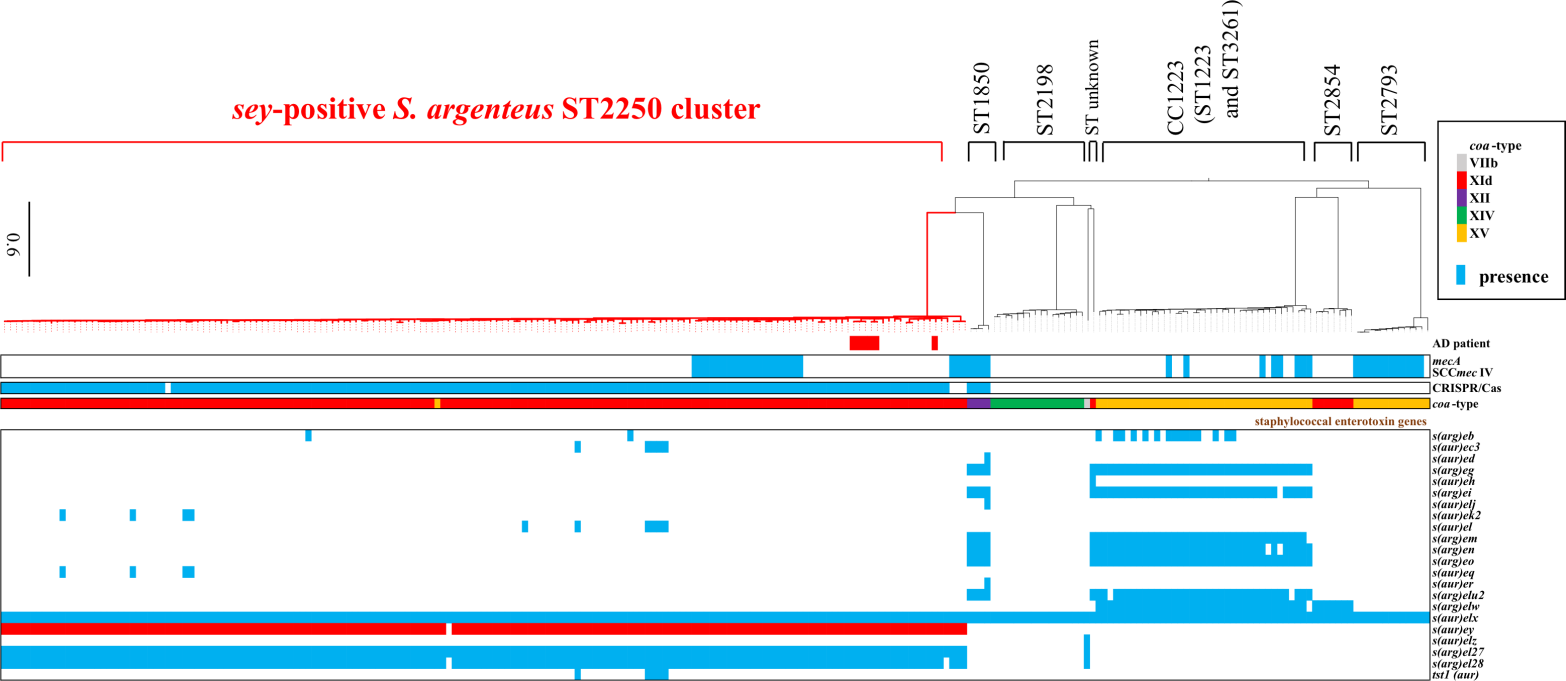
Phylogenetic analysis of *sey*-positive *S. argenteus* and staphylococcal enterotoxin gene profiles. A maximum likelihood phylogenetic tree of 244 *S*. *argenteus* strains with heatmaps indicating the metadata is shown. Only strains for which Illumina read data were available were analyzed. Metadata included AD patient isolates from Keio University in this study (Red), SCC*mec*IV cassette (Blue), CRISPR/Cas (Blue), coagulase type (*coa*-type), and staphylococcal enterotoxin (*ent*) genes. In the matrix of *ent* genes, blue indicates the presence of these genes and the presence of the *sey*-like gene is highlighted by red. The presence or absence of the toxin genes was determined using Abricate software v1.0.1. Parentheses in each cluster indicate the ST types of *S. argenteus*.

We isolated *S. argenteus* from 4 out of 145 patients with AD (2.8%) (Table S1). Through routine PCR analysis, we identified *sey* gene in six isolates of *S. argenteus* from four AD patients in Tokyo, Japan. These isolates tested positive for *sey* but not for other enterotoxins produced by *S. aureus*. We conducted whole-genome sequencing of 21 *S*. *argenteus* strains, including clinically isolated strains from Hokkaido and West Japan (19). The *S. argenteus* strain SARG0275, isolated from an AD patient, underwent complete sequencing through hybrid assembly using Illumina and Nanopore reads (Fig. 1). We identified the *sey*-like gene, encoding 221 amino acids, located on the chromosome of SARG0275 (Fig. 1). Examination of the boundary nucleotide sequences of the *sey* gene revealed inverted repeats of 7 bp sequences. SARG0275 had a chromosome size of 2,755,800 bp and one plasmid size of 2,243 bp. Multilocus sequence typing (MLST) analysis indicated that SARG0275 belonged to ST2250. Comparative analysis showed that *S. argenteus* SARG0275 shared ~80% structural similarity with *S. aureus* MW2 (Fig. S1). We have established a genomic and pathogenetic factors database, the “Japan Clone Library” of *S. aureus*, comprising strains clinically isolated from patients with various diseases at medical institutions in Japan (BioProject accession #PRJDB10779). These 197 strains represent 41 clusters identified by PFGE analysis from 3,000 *S*. *aureus* strains collected from various lesions or samples, including sepsis, pneumonia, TSS, impetigo, Staphylococcal Scalded Skin Syndrome, atopic dermatitis, and furunculosis, from approximately 3,000 patients. Population structure analysis of *S. argenteus* revealed that the six *S*. *argenteus* isolates from AD patients were ST2250 lineage, and close to clusters of *S. aureus* isolated from skin infections (Fig. S2). Pan-genome analysis, incorporating whole-genome sequences of *S. argenteus* strains from NCBI public data, identified seven distinct lineages [Clonal Complex (CC) 1223, ST1850, ST2198, ST2250, ST2793, ST2854, and ST unknown], with the largest lineage being ST2250 (Fig. 2). ST2250 was the only lineage positive for *sey*, while other ST types tested negative for it (Fig. 1 and 2). The six *S*. *argenteus* isolates isolated from AD patients in this study were *mecA* negative.

**Fig 3:**
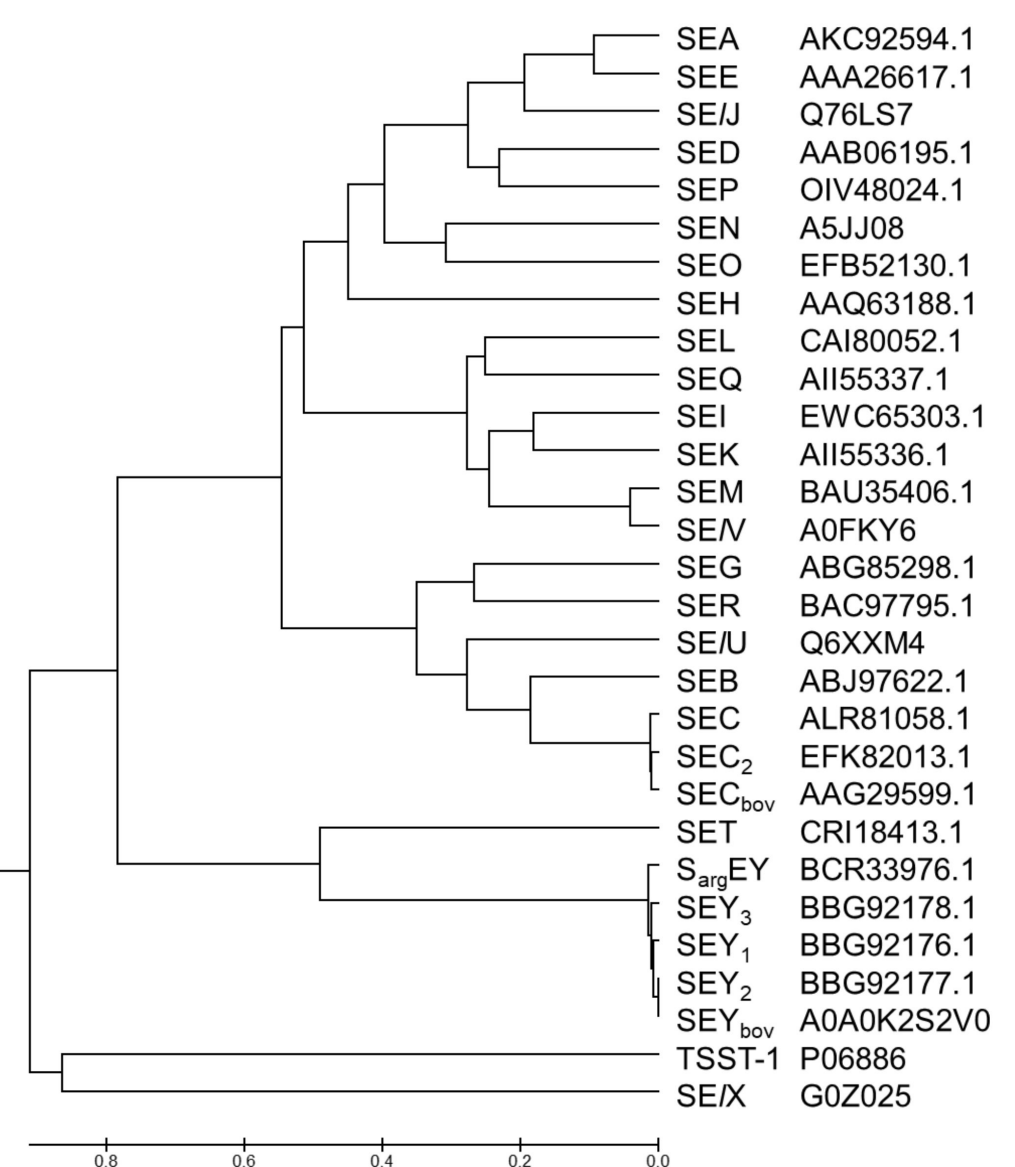
Phylogenetic tree of S_arg_EY and staphylococcal enterotoxin members in *S. aureus* with respective available accession numbers of the protein database. The tree was constructed by ClustalW alignment followed by the UPGMA method using Mega software.

The deduced amino acid sequence of SEY shared 98% sequence identity with the SEY_2_ sequence from *S. aureus*. Phylogenetic analysis (Fig. 3) revealed that SEY from *S. argenteus* clustered with *S. aureus* SEY variants. Specifically, the predicted mature SEY between *S. aureus* and *S. argenteus* exhibited three substituted amino acid residues (T40A, K63Q, and A161T), respectively.

We evaluated the emetic activity of *S. argenteus* SEY in an animal model. As illustrated in Table S4, two common marmosets exhibited emetic activity out of four animals tested, whereas no activity was observed in the PBS control group. In accordance with the guidelines of the International Nomenclature Committee for Staphylococcal Superantigens (20), we designated the open reading frame (ORF) of the *S. argenteus sey* gene as S_arg_EY gene. Recombinant S_arg_EY showed a deduced molecular mass of the mature toxin very similar to SEY_2_, approximately 23.5 kDa (Fig. S3, upper). We also confirmed the immunological cross-reaction of S_arg_EY against rabbit sera anti-SEY_2_ (Fig. S3, middle). Cross-reactive bands were also observed in SEY_2_ using mouse antisera raised against S_arg_EY (Fig. S3, lower). Both antisera were able to react to both SEY from *S. aureus* and *S. argenteus*, respectively, indicating cross-immunological reactivity between both toxins. Moreover, while SEY_bov_ reported instability against heat, pepsin, and trypsin treatment (21), SEY subtypes from human isolates demonstrated greater stability to heat treatment (22). We demonstrated that S_arg_EY exhibited similar characteristics to SEY variants, showing stability against heat treatment (Fig. S4).

### T-Cell stimulation activities of S_arg_EY

**Fig 4:**
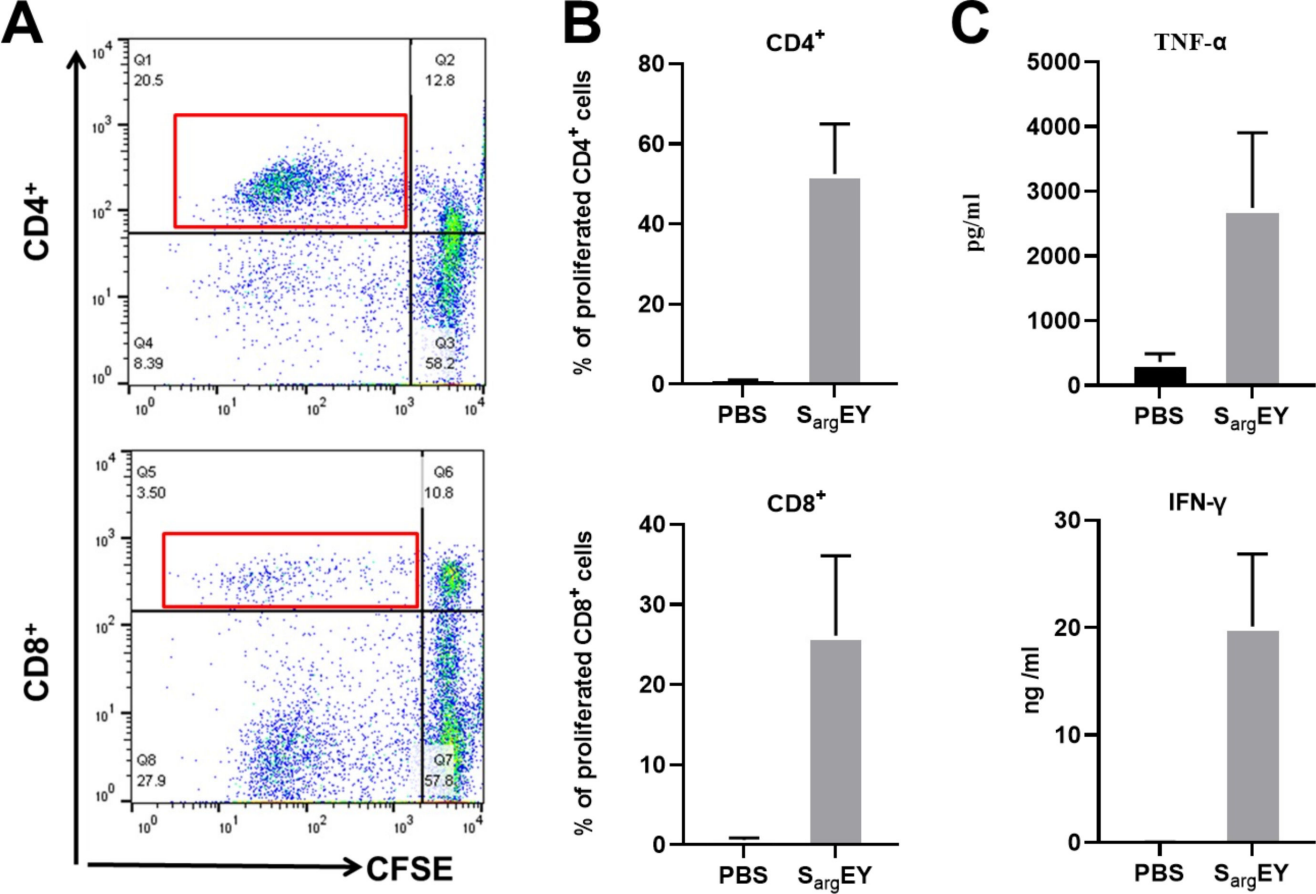
T-cell stimulation activities of S_arg_EY. CFSE-labeled human PBMCs were stimulated with 10 ng/mL of S_arg_EY or PBS for 5 days. Flow cytometry analysis was performed to evaluate the percentage of dividing T cells in response to S_arg_EY stimulation by gating CD4^+ high^ CSFE^low^ and CD8^+ high^ CSFE^low^ (red box) (**A**). The percentages of proliferating CD4^+^ and CD8^+^ T cells were calculated and compared with PBS as negative control (**B**). Cytokine production was assessed by ELISA of culture supernatant from PBMCs stimulated with the toxin or PBS (**C**). The bars represent the mean and standard errors in three to four experiments.

To assess S_arg_EY’s capacity to induce T-cell proliferation and cytokine production, human PBMCs were cultured in the presence or absence of S_arg_EY. Staphylococcal enterotoxin H (SEH) served as the positive control, and staphylococcal enterotoxin T (SET) was used as a structurally similar but phylogenetically distinct toxin from the SEY group. Consistent with our previous findings on SEY variants, flow cytometry revealed patterns indicative of dividing cells in both CD4^+^ and CD8^+^ T-cell populations cultured with S_arg_EY (Fig. 4A). As depicted in Fig. 4B, T-cell stimulation activity of S_arg_EY demonstrates substantial cell divisions in CD4^+^ and CD8^+^ T-cell subset populations following stimulation with the toxin. S_arg_EY induced the production of TNF-α and IFN-γ (Fig. 4C). These activities were comparable to those of SEY (22). T-cell proliferation was also confirmed following stimulation with recombinant staphylococcal enterotoxin H (23) and SET (Fig. S5). The results were analyzed statistically by two-tailed Student’s *t* test (22).

**Fig 5:**
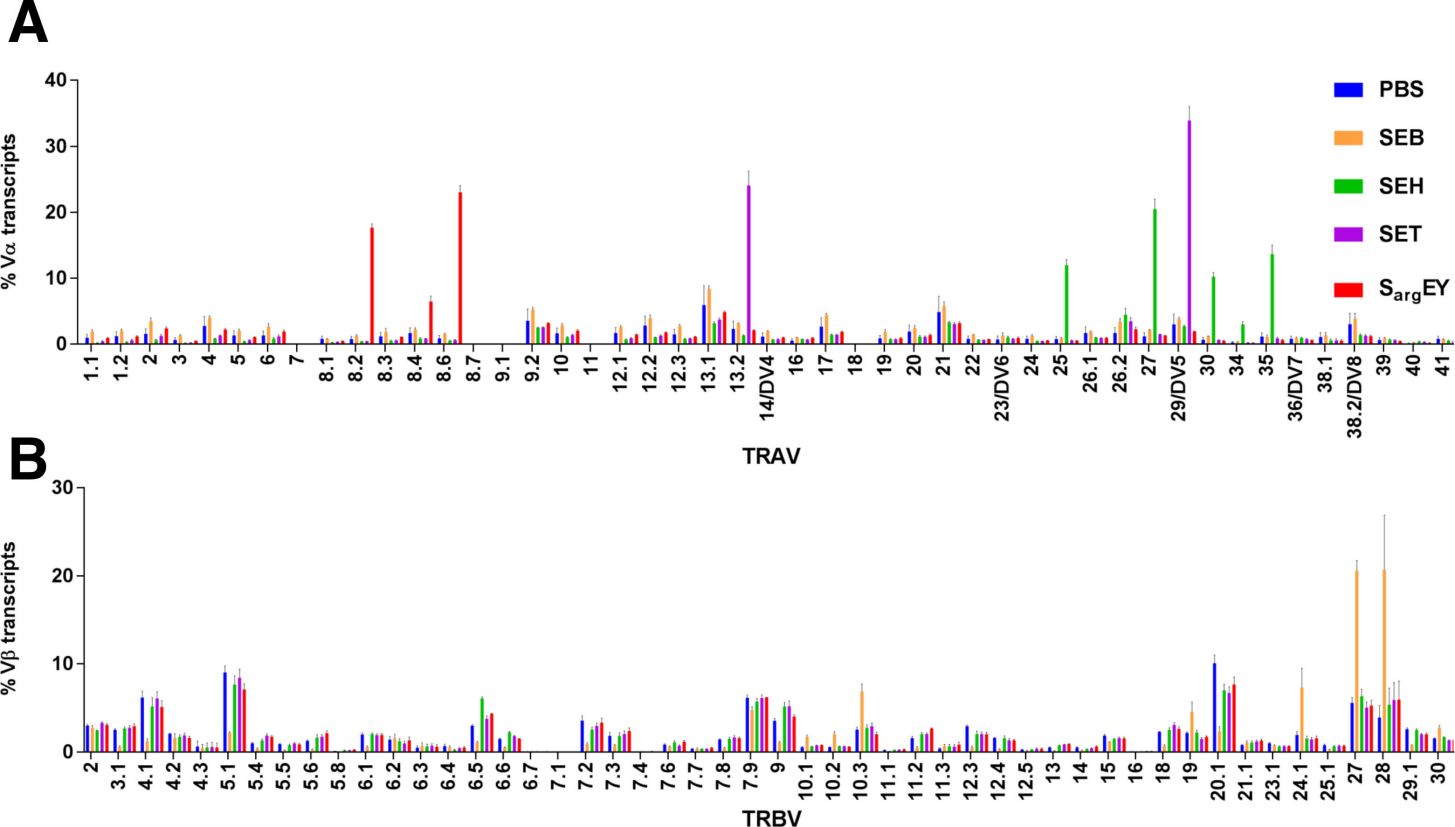
Analysis of TCR Vα/β sequencing from total RNA samples after *in vitro* stimulation of human PBMCs with 10 ng/ml of SEB, SEH, and S_arg_EY or 100 ng/mL SET. Transcripts from TRAV (**A**) and TRBV (**B**) gene usage. Data represent the mean values and SEM of three healthy donors.

Most superantigens are known to activate T cells via TCR Vβ, with SEH and SEY being exceptions, capable of activating them in a TCR Vα-dependent manner (22, 24). We demonstrated that SEY_2_ activated TRAV 8.2 and 8.6 by TCR sequencing. However, several superantigens, including SET, remain uncharacterized regarding their TCR Vα/β specificity (25). In this study, we applied a similar approach to determine TCR repertoires following T-cell stimulation with such uncharacterized superantigens. TCR sequencing revealed Vα-specific T-cell activation by S_arg_EY and SET (Fig. 5A). Notably, a significant enhancement of TCR Vα transcription was observed for each of these toxins: TRAV 8.2, 8.4, and 8.6 for S_arg_EY; and TRAV 13.2 and 29/DV5 genes for SET. We employed SEH as a positive control for TCR Vα activation, which is known to enhance TRAV 27 transcription as detected by real-time RT-PCR method (24). In addition to TRAV 27, our TCR sequencing confirmed an expansion of TRAV 25, 30, 34, and 35 gene transcripts in SEH-stimulated T cells. Conversely, following stimulation with staphylococcal enterotoxin B (SEB), enhanced expression of TRBV 10.3, 19, 24.1, 27, and 28 was observed (Fig. 5B), while no particular TRAV enhancement was noted (Fig. 5A). In contrast to these findings in humans, S_arg_EY exhibited little mitogenic activity to stimulate mouse splenocytes at any concentration, whereas SEA, even at 10 ng/mL, displayed strong activity in proliferating mouse cells (Fig. S6). Overall, these results clearly indicate that S_arg_EY, like SEY, exhibits superantigenic activity in humans but far less in mice.

## DISCUSSION

In this study, we report the first detection of *sey*-positive community-associated *S. argenteus* isolated from four patients with atopic dermatitis in Japan. The detection rate of *S. argenteus* (2.8%) was lower than that of *S. aureus* (57.9%) in AD patients (13). Previously, we reported *sey*-positive *S. aureus* lineages, including CC20, CC59, and CC121, isolated from skin and soft tissue infections, documented in the Japan Clone Library (22). Conversely, *S. argenteus* comprises seven clusters (CC1223, ST1850, CC2198, ST2250, ST2793, ST2854, and ST unknown) based on phylogenetic tree analysis thus far (26). Notably, we found that ST2250 was the sole lineage positive for *sey*. Sequence data indicated that *s*arg*ey* is sandwiched by a 7-base direct repeat, suggesting that it is horizontally acquired by ST2250. The origin of the *s*arg*ey* gene in *S. argenteus* remains elusive. The mobile element of *s*arg*ey* did not possess any DNA recognition motif of *hsd* in the restriction-modification (R-M) system, suggesting that the R-M system is not a selection trait to acquire *s*arg*ey* gene by ST2250 (Table S5). Ohnishi and Miyoshi-Akiyama et al. reported the first case in Japan where this clone was isolated from pediatric purulent lymphadenitis (27, 28). Furthermore, two cases of food poisoning by *seb*-positive *S. argenteus* ST1223 have been documented in Tokyo, Japan (29, 30). Pan-genome phylogenetic analysis indicates that ST1223 possesses several enterotoxin genes, including the classical enterotoxin gene *seb* (Fig. 2). The presence of the *seb* gene on the *S. argenteus* genomic island SargPI, similar to the *S. aureus* pathogenicity island SaPIIshikawa11 (31), likely contributes to the increased virulence of the ST1223 *S. argenteus* lineage. In a recent report, *S. argenteus* CC2250 and CC1223 were isolated from various retail foods in coastal cities of South China (32), suggesting that these lineages could potentially cause food intoxication.

Hansen et al. reported that all methicillin-resistant *S. argenteus* (MRSArg) carried a SCC*mec*IV cassette (26). In this study, MRSArg was not detected in Japan (Table S2).

We characterized a superantigen transcribed by *S. argenteus* SARG0275 from an AD patient. Our results revealed that S_arg_EY shares high similarity with *S. aureus* SEY, particularly in terms of mitogenic activity, cross-immunology, stability characteristics, and emetic activity. Our findings demonstrated that S_arg_EY is a potent inducer of T-cell proliferation and inflammatory cytokine production in human PBMCs. Superantigens, defined as exotoxins with a specific mechanism of T-cell stimulation, can induce massive proliferation in both CD4^+^ and CD8^+^ T cells (33). Superantigen-stimulated T cells also produce substantial levels of cytokines and chemokines, especially interleukin-1 (IL-1), IL-2, IFN-γ, and TNF-α (34, 35). The properties of S_arg_EY described in this study align well with those of a typical superantigen. Moreover, superantigens are produced by a variety of microorganisms, mainly in the Staphylococcaceae family (36), and play important roles in the establishment of bacterial infection and/or colonization by modifying the environment of the infection site and altering the local T-cell population to elicit effective inflammatory responses (34). As a consequence of superantigen exposure, the immune system’s entire inactivation provides the microbes with an advantageous environment for rapid growth in the host (33).

*S. argenteus* isolates of ST2250 have been identified as a globally dominant clone, acquiring various exotoxins and antibiotic resistance genes from livestock-associated *S. aureus* (1). Additionally, ST2250 carrying S_arg_EY gene has been reported from diverse sources such as human nasal swabs (3), cases of bacteremia (8), and pork (37). Similar to S_arg_EY, we previously demonstrated that SEY subtypes 1, 2, and 3 were also encoded on the chromosome. The high similarity in the deduced amino acid sequences of these *S. aureus* SEY variants and S_arg_EY, combined with the fact that both *sey* genes are embedded in the chromosome, suggests a common ancestral origin.

In the present study, we elucidated that S_arg_EY and SET activate human T cells through Vα-specific stimulation. It is noteworthy that SEY and S_arg_EY exhibit the closest similarity, with a 32% identity of the SET amino acid sequence among *S. aureus* SE members. Despite this, their TCR Vα specificity for T-cell proliferation differs significantly. We also observed slight differences in the TCR Vα specificity of S_arg_EY compared to SEY_2_, although they predominantly activated TRAV 8.2 and 8.6. In addition to SEH, our study contributes to defining detailed TCR Vα activation profiles of newly identified staphylococcal superantigens. Furthermore, TCR sequencing revealed other previously undescribed Vα repertoires induced by SEH (Fig. 5A). TCR sequencing for the characterization of specific Vα and Vβ expansions following superantigen stimulation should be considered, addressing limitations of flow cytometry and PCR-based methods.

Recent reports have highlighted the global distribution and implications of *S. argenteus* in causing human infections and food poisoning (6, 29). Apart from its superantigenic character, SEY_bov_ has been reported to exhibit emetic activity in house musk shrews and common marmosets, indicating its potential to cause food poisoning (21, 38). Suzuki et al. (29) reported *S. argenteus* isolated from a food poisoning outbreak in Tokyo in 2010, which produced S_arg_EB. In our study, S_arg_EY demonstrated emetic activity in a primate model and exhibited protein stability to heat treatment. Therefore, S_arg_EY could be presumed to have the potential to cause food poisoning.

We demonstrated that *Staphylococcus* from AD patients possess SEY or S_arg_EY. Both *S. aureus* and *S. argenteus* commonly adhere to lesions, and prolonged infection may exacerbate the clinical condition. Additionally, our study uncovered the biological characteristics of S_arg_EY transcribed in *S. argenteus*, which were similar to their counterparts in *S. aureus* in many aspects. Further research is needed to determine the implications of SEY and S_arg_EY in causing staphylococcal diseases and their clinical significance. The biological characteristics of S_arg_EY gene harbored by ST2250 in our study suggest their potential pathogenicity in *S. argenteus* as a recently emerging pathogen in AD patients.

## MATERIALS AND METHODS

### Bacterial isolates and culture conditions

Six *S*. *argenteus* isolates were obtained from patients with atopic dermatitis at Keio University Hospital between 2017 and 2019, while five were sourced from healthy individuals and patients with otorrhea at Kochi University Hospital. Additionally, two isolates were collected from a previous study (Table S1). Ethical approval for the protocol was obtained from the Keio University School of Medicine Ethics Committee (approval number 20130384), Hiroshima University Ethics Committee (approval number E-412), and the National Institute of Infectious Diseases (NIID) Ethics Committee (approval number 1338). These strains were preserved in 15% glycerol stock at −80°C. Both *S. argenteus* and *S. aureus* isolates were cultured routinely in tryptic soy broth (TSB; BD Microbiology System, MD, USA) at 37°C overnight with aeration in a water bath shaker or on tryptic soy agar plates before the commencement of experiments. Genomic DNA extraction was performed as previously described (39).

### Staphylococcal enterotoxin gene detection

A multiplex primer set was utilized to detect SEs genes in both *S. aureus* and *S. argenteus*, including staphylococcal enterotoxin A (SEA), SEB, SEC, SED, SEE, SEG, SEH, SEI, SE*l*J, SEK, SEL, SEM, SEN, SEO, SEP, SEQ, SER, and SEY, along with toxic shock syndrome toxin-1 (TSST-1), using a previously described method (8).

### Genome sequencing and assembly

Genomic DNA was extracted using the QIAamp DNA purification kit (QIAGEN) following the manufacturer’s instructions. DNA libraries were prepared for sequencing using Enzymatics 5× WGS reagents (BioStream Co., Ltd) and subsequently pooled. DNA sequencing was conducted on the Illumina HiSeq X FIVE platform at Macrogen Japan Corporation (Tokyo, Japan). Raw reads were assembled using Shovill v1.0.9 (available at https://github.com/tseemann/shovill) with default settings. A single colony was selected from a Luria-Bertani (LB) agar plate for overnight culture in LB broth at 37°C. For Nanopore sequencing, genomic DNA was extracted using the Qiagen Genomic-tip 20/G Kit (Qiagen). Long-read library preparation for MinION [Oxford Nanopore Technologies (ONT)] was carried out using the SQK-RBK004 Rapid Barcoding Kit (ONT) without DNA size selection, and sequencing was performed using MinKNOW software with a FLO-MIN106 R9.4 flow cell (ONT). Fast5 read files were base called and demultiplexed with Guppy v4.0.15 (ONT). Hybrid assembly of Illumina short reads and MinION long reads was conducted using the Unicycler v0.4.8 hybrid assembler (40) with default parameters. The Unicycler pipeline automatically identified and trimmed overlaps for circular genomes and oriented the genome to start with the dnaA gene. Default parameters were used for all software unless otherwise specified.

### Phylogenetic tree analysis

Assembled reads underwent annotation using Prokka v1.14.6 (41) or the DFAST-core pipeline v1.2.11 (42). Pan-genome analysis was conducted using PEPPA (https://github.com/zheminzhou/PEPPAN) (43) with default settings. Core SNP sites were extracted using *snippy-core* with default settings, and recombinogenic SNP sites were subsequently removed using Gubbins v2.3.4 (44). A maximum likelihood (ML) tree was constructed with RAxML-NG v0.9.0 (available at https://github.com/amkozlov/raxml-ng) (45) based on the generalized time reversible (GTR) model of nucleotide substitution with among-site rate heterogeneity across four categories, ascertainment bias correction (LEWIS), and 100 bootstrap replicates. Phylogenetic trees were visualized using Phandango (https://jameshadfield.github.io/phandango/#/) (46) and FigTree v1.4.4 (available at http://tree.bio.ed.ac.uk/software/figtree/), with subsequent editing in Graphic for Mac v3.0 (https://www.graphic.com). Sample list for phylogenetic tree analysis of *S. argenteus* was summarized in Table S2.

### Cloning of toxins

The gene fragments corresponding to the predicted mature forms of S_arg_EY, SEH, and SET were amplified using KOD-Plus-Neo (Toyobo, Osaka, Japan). The primer pairs used are listed in Table S3. PCR products were purified, digested with restriction enzymes, and ligated into pET 22b+ for S_arg_EY and SET or pET 28a for SEH as cloning and expression vectors (both plasmids from Novagen, Madison, USA). The plasmid constructs were transformed into *Escherichia coli* DH5α, and gene integrity was confirmed by DNA sequencing using BigDye Terminator V 3.1 (ABI 3100xl: Applied Biosystems, USA). Recombinant toxins were expressed in *E. coli* strain BL21(DE3).

### Expression of recombinant toxins

Bacteria were cultured at 37°C in 300 mL Luria-Bertani medium containing 100 µg/mL ampicillin until reaching an optical density (OD) of 0.5–0.6 at 600 nm. Expression of the inserted gene was induced with 0.5 mM IPTG (isopropyl-1-thio-β-d-galactopyranoside; Nacalai Tesque, Kyoto, Japan), followed by incubation at 30°C for 24 h as previously described (39). Harvested cells were lysed by sonication in lysis buffer (5 mM imidazole, 50 mM NaH_2_PO_4_, and 300 mM NaCl, pH 7.0) on ice. The cells were collected by centrifugation (9,000 × *g* at 4°C for 20 min), and recombinant toxins were purified using Talon-Cobalt affinity chromatography (Clontech Laboratories, Inc.). Protein purity was analyzed by 12% polyacrylamide gel electrophoresis. Purified protein was dialyzed against 1,000 times its volume in Dulbecco’s phosphate-buffered saline (2.68 mM KCl, 1.46 mM KH_2_PO_4_, 136.9 mM NaCl, 8 mM Na_2_HPO_4_·12H_2_O, pH 7.4) at 4°C for 24 h. Endotoxin contamination in toxins was removed using EndoTrap (Hyglos, Bernried, Germany). Protein concentration was measured using the Bio-Rad protein assay (Bio-Rad, Hercules, CA, USA) with bovine serum albumin (BSA; Sigma-Aldrich, St. Louis, USA) as the standard protein.

### Preparation of human T-cell proliferation and cytokine production

*In vitro* stimulation of PBMCs with toxins was conducted following established protocols. Briefly, 2 × 10^6^ PBMCs were labeled with 5-(and -6)-carboxyfluorescein diacetate succinimidyl ester (CFSE; Tonbo Biosciences, San Diego, CA, USA) in 1 mL complete RPMI-1640 medium (RPMI-1640 supplemented with 2.5% fetal calf serum and 1% penicillin/streptomycin) and were stimulated with 10 ng/mL of S_arg_EY. After a 5-day culture period, approximately 0.2 × 10^6^ cells were stained with PE-labeled anti-CD4 (Clone: RPA-T4, TONBO) and PE-Cy7-labeled anti-CD8a (Clone: RPA-T8, TONBO) antibodies and analyzed using a JSAN flow cytometer (Bay Biosciences, Kobe, Japan). The percentage of proliferating cells was determined by gating onto CD4^+^CFSE^low^ cells or CD8^+^CFSE^low^ cells, using FlowJo software (BD Biosciences). For cytokine production analysis, around 2 × 10^6^ human PBMCs were stimulated with 10 ng/mL of S_arg_EY in complete RPMI-1640 medium. After a 5-day incubation, culture supernatants were collected, and TNF-α and IFN-γ levels were measured by ELISA (Invitrogen) following the manufacturer’s instructions.

### TCR sequencing

Human PBMCs (4 × 10^6^ cells) were stimulated for 5 days with 10 ng of SEB (Sigma-Aldrich, Tokyo, Japan), SEH, and S_arg_EY, or 100 ng of SET, and then the cells were collected in 350 µL RLT lysis buffer (Qiagen, Hilden, Germany). Total RNA was extracted using the RNEasy mini kit (Qiagen, Hilden, Germany). TCR libraries were prepared using the SMARTer Human TCR α/β profiling kit (Takara Bio, CA, USA) following the manufacturer’s instructions. Purified products were sequenced on an Illumina MiSeq instrument (Illumina, San Diego, CA, USA). Analysis of TCR sequences was carried out using MiXCR software (47) for raw data processing with CDR3 extraction and gene alignment, tcR (48), and VDJtools software (49) for comparing TCR repertoires of each sample.

### Emetic activity test

The emetic activity of S_arg_EY was evaluated in common marmosets, a recently established emetic primate model (38). After a 16-h fast, animals were anesthetized using a combination of medetomidine (80 µg/kg; ZENOAQ, Fukushima, Japan) and midazolam (400 µg/kg; SANDOZ, Tokyo, Japan) via intramuscular injection. Approximately 1.5 mL of S_arg_EY in sterile PBS (final dose 250 µg/kg) was administered to common marmosets via orogastric intubation. For rapid recovery, the animals were intramuscularly administered atipamezole (320 µg/kg, ZENOAQ). Emetic responses, latency period of the first emetic response, and behavioral changes were evaluated based on 5-h video recordings.

## Data Availability

The chromosome and plasmid sequences of SARG0275 have been deposited at the DNA Data Bank of Japan (DDBJ) and ENA/Genbank under the accession numbers AP024410 and AP024411.

## SUPPLEMENTAL MATERIAL

Supplemental figures msphere.00505-24-s0001.pdf Fig. S1 to S6.

Supplemental references msphere.00505-24-s0002.docx References for supplemental material.

Legends msphere.00505-24-s0003.docx Supplemental figure legends.

Table 1 msphere.00505-24-s0004.pdf Strains and plasmid used in this study.

Table 2 msphere.00505-24-s0005.pdf Sample list for phylogenetic tree analysis based on the whole genome sequences of *S*. *argenteus.*

Table 3 msphere.00505-24-s0006.pdf Primer used for toxins gene cloning.

Table 4 msphere.00505-24-s0007.pdf Emetic activity test in monkey model.

Table 5 msphere.00505-24-s0008.pdf Recognition motifs of HsdS or HsdM in the type I-IV restriction-modification system of the *S*. *argenteus* isolates used in this study.

ASM does not own the copyrights to Supplemental Material that may be linked to, or accessed through, an article. The authors have granted ASM a non-exclusive, world-wide license to publish the Supplemental Material files. Please contact the corresponding author directly for reuse.
