# Supplementary material for: Genomic analysis and identification of a novel superantigen, SargEY, in Staphylococcus argenteus isolated from atopic dermatitis lesions
Source: mSphere. 2024 Jul 11;9(7):e00505-24. doi: 10.1128/msphere.00505-24 (PMC11288046; doi:10.1128/msphere.00505-24)
Supplement: Supplemental references — References for supplemental material. [file msphere.00505-24-s0002.docx]

**References of Supplemental Material**

1. Ohtsubo Y, Ikeda-Ohtsubo W, Nagata Y, Tsuda M. 2008. GenomeMatcher: a graphical user interface for DNA sequence comparison. BMC Bioinformatics 9:376. https://doi.org/10.1186/1471-2105-9-376
2. Page AJ, Cummins CA, Hunt M, Wong VK, Reuter S, Holden MT, Fookes M, Faush D, Keane JA, Parkhill J. 2015. Roary: rapid large-scale prokaryote pan genome analysis. Bioinformatics 31:3691-3693. <https://doi.org/10.1093/bioinformatics/btv421>
3. Stamatakis A. 2014. RAxML version 8: a tool for phylogenetic analysis and post-analysis of large phylogenies. Bioinformatics 30:1312-1312. <https://doi.org/10.1093/bioinformatics/btu033>
4. Suzuki Y, Kubota H, Ono HK, Kobayashi M, Murauchi K, Kato R, Hirai A, Sadamasu K. 2017. Food poisoning outbreak in Tokyo, Japan caused by Staphylococcus argenteus. Int J Food Microbiol 262:31-37. https://doi.org/ 10.1016/j.ijfoodmicro.2017.09.005
5. Ohnishi T, Shinjoh M, Ohara H, Kawai T, Kamimaki I, Mizushima R, Kamada K, Itakura Y, Iguchi S, Uzawa Y, Yoshida A, Kikuchi K. 2018. Purulent lymphadenitis caused by Staphylococcus argenteus, representing the first Japanese case of Staphylococcus argenteus (multilocus sequence type 2250) infection in a 12-year-old boy. J Infect Chemother 24:925-927. <https://doi.org/10.1016/j.jiac.2018.03.018>
6. Miyoshi-Akiyama T, Ohnishi T, Shinjoh M, Ohara H, Kawai T, Kamimaki I, Mizushima R, Kamada K, Itakura Y, Iguchi S, Uzawa Y, Yoshida A, Kikuchi K, Takemoto N. 2019. Complete Genome Sequences of Staphylococcus argenteus TWCC 58113, Which Bears Two Plasmids. Microbiol Resour Announc 8:e01582-18. <https://doi.org/10.1128/MRA.01582-18>
7. Holt DC, Holden MT, Tong SY, Castillo-Ramirez S, Clarke L, Quail MA, Currie BJ, Parkhill J, Bentley SD, Feil EJ, Giffard PM. A very early-branching Staphylococcus aureus lineage lacking the carotenoid pigment staphyloxanthin. Genome Biol Evol 3:881-895. <https://doi.org/10.1093/gbe/evr078>
8. Hansen TA, Bartels MD, Hogh SV, Dons LE, Pedersen M, Jensen TG, Kemp M, Skov MN, Gumpert H, Worning P, Westh H. 2017. Whole Genome Sequencing of Danish Staphylococcus argenteus Reveals a Genetically Diverse Collection with Clear Separation from Staphylococcus aureus. Front Microbiol 8:1512. <https://doi.org/10.3389/fmicb.2017.01512>
9. Soderquist B, Wildeman P, Stenmark B, Stegger M. 2020. Staphylococcus argenteus as an etiological agent of prosthetic hip joint infection: a case presentation. J Bone Jt Infect 6:172-175. <https://doi.org/10.7150/jbji.44848>
10. Tong SY, Schaumburg F, Ellington MJ, Corander J, Pichon B, Leendertz F, Bentley SD, Parkhill J, Holt DC, Peters G, Giffard PM. 2015. Novel staphylococcal species that form part of a Staphylococcus aureus-related complex: the non-pigmented Staphylococcus argenteus sp. nov. and the non-human primate-associated Staphylococcus schweitzeri sp. Nov. Int J Syst Evol Microbiol 65:15-22. <https://doi.org/10.1099/ijs.0.062752-0>
11. Zhang DF, Yang XY, Zhang J, Qin X, Huang X, Cui Y, Zhou M, Shi C, French NP, Shi X. 2018. Identification and characterization of two novel superantigens among Staphylococcus aureus complex. Int J Med Microbiol 308:438-446. <https://doi.org/10.1016/j.ijmm.2018.03.002>
12. Moradigaravand D, Jamrozy D, Mostowy R, Anderson A, Nickerson EK, Thaipadungpanit J, Wuthiekanun V, Limmathurotsakul D, Tandhavanant S, Wikraiphat C, Wongsuvan G, Teerawattanasook N, Jutrakul Y, Srisurat N, Chaimanee P, Eoin West T, Blane B, Parkhill J, Chantratita N, Peacock SJ. 2017. Evolution of the Staphylococcus argenteus ST2250 Clone in Northeastern Thailand Is Linked with the Acquisition of Livestock-Associated Staphylococcal Genes. mBio 8:e00802-17. <https://doi.org/10.1128/mBio.00802-17>
13. Zhang DF, Zhi XY, Zhang J, Paoli GC, Cui Y, Shi C, Shi X. 2017. Preliminary comparative genomics revealed pathogenic potential and international spread of Staphylococcus argenteus. BMC Genomics 18:808. <https://doi.org/10.1186/s12864-017-4149-9>
14. Tang Hallback E, Karami N, Adlerberth I, Cardew S, Ohlen M, Engstrom Jakobsson H, Svensson Stadler L. 2018. Methicillin-resistant Staphylococcus argenteus misidentified as methicillin-resistant Staphylococcus aureus emerging in western Sweden. J Med Microbiol 67:968-971. <https://doi.org/10.1099/jmm.0.000760>
15. Kaden R, Engstrand L, Rautelin H, Johansson C. 2018. Which methods are appropriate for the detection of Staphylococcus argenteus and is it worthwhile to distinguish S. argenteus from S. aureus? Infect Drug Resist 11:2335-2344. <https://doi.org/10.2147/IDR.S179390>
16. Schuster D, Rickmeyer J, Gajdiss M, Thye T, Lorenzen S, Reif M, Josten M, Szekat C, Melo LD, Schmithausen RM, Liegeois F, Sahl HG, Gonzalez JJ, Nagel M, Bierbaum G. 2017. Differentiation of Staphylococcus argenteus (formerly: Staphylococcus aureus clonal complex 75) by mass spectrometry from S. aureus using the first strain isolated from a wild African great ape Int J Med Microbiol 307:57-63. <https://doi.org/10.1016/j.ijmm.2016.11.003>
17. Jiang B, You B, Tan L, Yu S, Li H, Bai G, Li S, Rao X, Xie Z, Shi X, Peng Y, Hu X. 2018. Clinical Staphylococcus argenteus Develops to Small Colony Variants to Promote Persistent Infection. Front Microbiol 9:1347. <https://doi.org/10.3389/fmicb.2018.01347>
18. Diot A, Dyon-Tafani V, Bergot M, Tasse J, Martins-Simoes P, Josse J, Valour F, Laurent F. 2020. Investigation of a Staphylococcus argenteus Strain Involved in a Chronic Prosthetic-Joint Infection. Int J Mol Sci 21:6245. <https://doi.org/10.3390/ijms21176245>
19. Neyaz L, Karki AB, Fakhr MK. 2019. The Whole-Genome Sequence of Plasmid-Bearing Staphylococcus argenteus Strain B3-25B from Retail Beef Liver Encodes the Type VII Secretion System and Several Virulence Factors. Microbiol Resour Announc 8:e00962-19. <https://doi.org/10.1128/MRA.00962-19>
20. Aung MS, Urushibara N, Kawaguchiya M, Sumi A, Takahashi S, Ike M, Ito M, Habadera S, Kobayashi N. 2019. Molecular Epidemiological Characterization of Staphylococcus argenteus Clinical Isolates in Japan: Identification of Three Clones (ST1223, ST2198, and ST2550) and a Novel Staphylocoagulase Genotype XV. Microorganisms 7:389. <https://doi.org/10.3390/microorganisms7100389>
21. Kitagawa H, Ohge H, Hisatsune J, Masuda K, Aziz F, Hara T, Kuroo Y, Sugai M. 2019. Low incidence of *Staphylococcus argenteus* bacteremia in Hiroshima, Japan. J Infect Chemother 26:140-143. https://doi.org/10.1016/j.jiac.2019.07.011
