# Supplementary material for: Genomic analysis and identification of a novel superantigen, SargEY, in Staphylococcus argenteus isolated from atopic dermatitis lesions
Source: mSphere. 2024 Jul 11;9(7):e00505-24. doi: 10.1128/msphere.00505-24 (PMC11288046; doi:10.1128/msphere.00505-24)
Supplement: Legends — Supplemental figure legends. [file msphere.00505-24-s0003.docx]

**Supplemental Material　Figure legends**

**Figure S1.** Dot plot comparing the chromosomes of *S. argenteus* SARG0275 and *S. aureus* MW2, revealing structural similarity with homology ranging from 70% to 80%. Sequence comparison and visualization were conducted using GenomeMatcher v3.0.6 (1).

**Figure S2.** Phylogenetic relationships among 197 human clinical *S. aureus* isolates from the Japan clone library and six *S. argenteus* isolates from AD patients. A core-genome alignment was generated using the Roary pipeline (2), followed by construction of a maximum likelihood tree using RAxML (with the "-m GTRGAMMA" option) (3). The analysis illustrates that *S. argenteus* from AD patient skin falls within the cluster of *S. aureus* causing skin infections. The color box legend indicates the diagnosis of the patient whose sample was isolated. Grey denotes no information is available.

**Figure S3.** Investigation of antigenic sharing and native protein by immunoassay. One hundred nanograms of purified SEY_2_ and S_arg_EY per well in 12% polyacrylamide gel were stained with Coomassie blue (**Uppe**r). The proteins from another gel were transferred onto nitrocellulose membrane, and signals were detected using immunized rabbit sera anti-SEY_2_ (**Middle**) and mice sera anti-S_arg_EY (**Lower**).

**Figure S4.** Comparison of S_arg_EY and BSA stability against heat and digestive enzyme treatments. S_arg_EY and BSA samples (indicated by arrows) were prepared at a concentration of 100 µg/ml in PBS. The samples were subjected to the following treatments: heated at 100 °C (A), treated with pepsin at 100 µg/mL (B), or treated with trypsin at 50 µg/mL (C). Samples were collected at specified times and analyzed using a 12% polyacrylamide gel under denaturing conditions. Abbreviations: NT, no treatment; P, pepsin; T, trypsin. The BSA sample data was taken from a previously published study (22).

**Figure S5.** Mitogenic activity of SEH and SET in human PBMCs. Approximately 2 × 10^6^ CFSE-labeled PBMCs in 1 ml complete RPMI-1640 medium were stimulated with indicated toxins. The percentage of proliferated cell division of CD4^+^ and CD8^+^ T cells following 5 days of 0.1 to 100 ng/ml SEH and SET stimulation was analyzed by flow cytometry. The line graph shows the mean data from 3 healthy donors with standard error mean. The legend indicates PBS or toxins.

**Figure S6.** Comparison of SEA, SEY_2_, and S_arg_EY mitogenic activity in mice splenocytes. Splenocytes were stimulated with various concentrations of toxins and BSA as a protein control for 72 h of incubation. Cell proliferation was determined by the CCK-8 assay. Each bar represents the mean standard error means for triplicate assays from a representative experiment. These data were assayed in three experiments.
