# Supplementary material for: Genomic analysis and identification of a novel superantigen, SargEY, in Staphylococcus argenteus isolated from atopic dermatitis lesions
Source: mSphere. 2024 Jul 11;9(7):e00505-24. doi: 10.1128/msphere.00505-24 (PMC11288046; doi:10.1128/msphere.00505-24)
Supplement: Table 1 — Strains and plasmid used in this study. [file msphere.00505-24-s0004.pdf]

Supplementary Table 1. Strains and plasmid were used in this study.

Strain or plasmid	Purpose or characteristics	Source or reference
E.coli		
DH5α	Cloning strain	from Takara Bio Inc.
BL-21(DE3)	Host expression for recombinant protein	from Merck
S. argenteus		
SARG0275	*sey*$^{+}$, ST2250, *sey* cloning, isolated from AD patient K029	This study
SARG1271	*sey*$^{+}$, ST2250, *sey* cloning, isolated from AD patient K071	This study
SARG1913	*sey*$^{+}$, ST2250, *sey* cloning, isolated from AD patient K071	This study
SARG2343	*sey*$^{+}$, ST2250, *sey* cloning, isolated from AD patient K064	This study
SARG3091	*sey*$^{+}$, ST2250, *sey* cloning, isolated from AD patient K071	This study
SARG5253	*sey*$^{+}$, ST2250, *sey* cloning, isolated from AD patient K224	This study
JH5333	*sey*$^{+}$, ST2250, healthy nasal	This study
JH5336	*sey*$^{-}$, ST1223, otitis, isolated from otorrhea	This study
JH5337	*sey*$^{-}$, ST1223, otitis, isolated from otorrhea	This study
JH5338	*sey*$^{+}$, ST2250, otitis, isolated from otorrhea	This study
JH5340	*sey*$^{-}$, ST1223, healthy nasal	This study
JH6199	*sey*$^{+}$, ST2250, cellulitis	1)
JH6211	*sey*$^{+}$, ST2250, carther-related blood stream infection	1)
S. aureus		
No.10	*seh*$^{+}$, CC81, *seh* cloning	2)
Fukuoka 5	*set*$^{+}$, food poisoning, *set* cloning	3)

REFERENCE:
1) Kitagawa H, et al., 2019. Low incidence of Staphylococcus argenteus bacteremia in Hiroshima, Japan J Infect Chemother. 26(1):140-143.
2) Sato'o Y. et al., 2015. Positive Regulation of Staphylococcal Enterotoxin H by Rot (Repressor of Toxin) Protein and Its Importance in Clonal Complex 81 Subtype 1 Lineage-Related Food Poisoning. Appl Envirn Microbiol. 81(22):7782-7790.
3) Ono K. H. et al., 2008. Identification and characterization of two novel staphylococcal enterotoxins, types S and T. Infect Immun. 76(11):4999-5005.
