# Supplementary material for: Genomic analysis and identification of a novel superantigen, SargEY, in Staphylococcus argenteus isolated from atopic dermatitis lesions
Source: mSphere. 2024 Jul 11;9(7):e00505-24. doi: 10.1128/msphere.00505-24 (PMC11288046; doi:10.1128/msphere.00505-24)
Supplement: Table 2 — Sample list for phylogenetic tree analysis based on the whole genome sequences of S. argenteus. [file msphere.00505-24-s0005.pdf]

Supplementary Table 2. Sample list for phylogenetic tree analysis based on the whole genome sequences of *S. argenteus*.

BioProject	BioSample	Strain	#Organism Name	Assembly	Level	(Sequence Reads Achaive) RUN	collection date	country	ST	*mecA*	host disease	isolation source	Reference
PRJDB10984	SAMD00269395	JARB-HU0072 (P102)	*Staphylococcus argenteus*	-	SRA			Japan	ST2198	-	soft skin tissue infection (open pus)	human skin	This study
PRJDB10984	SAMD00269575	JARB-HU0252 (P396)	*Staphylococcus argenteus*	-	SRA			Japan	ST2250	-	soft skin tissue infection (non-open pus)	human skin	This study
PRJDB5453	SAMD00071154	Tokyo13064	*Staphylococcus argenteus*	-	SRA	DRR083591	2010-08	Japan	ST1223	-	-	food poisoning	1
PRJDB5453	SAMD00071155	Tokyo13069	*Staphylococcus argenteus*	-	SRA	DRR083592	2010-08	Japan	ST1223	-	-	food poisoning	1
PRJDB6867	SAMD00114619	58113	*Staphylococcus argenteus*	GCA_003967115.1	Complete	-	2018	Japan	ST2250	-	-	-	2
PRJDB7256	SAMD00133958	SARG0275	*Staphylococcus argenteus*	GCA_005397745.1	Contig	-	2016-11-28	Japan	ST2250	-	atopic dermatitis	human skin (upper back)	This study
PRJDB7731	SAMD00153157	TWCC58113	*Staphylococcus argenteus*	-	SRA	DRR161060-DRR161061	2017	Japan	ST2250	-	-	-	3
PRJDB9562	SAMD00215874	TUM19485	*Staphylococcus argenteus*	GCA_012862375.1	Contig	-	2019-12-27	Japan	ST1223	-	-	-	
PRJEA62885	SAMEA2272597	MSHR1132	*Staphylococcus argenteus*	GCA_000236925.1	Complete	-	Sep-2006	Australia	ST1850	+	necrotizing fasciitis	blood	4
PRJEB1915	SAMEA2445518	2588STDY5763869	*Staphylococcus argenteus*	GCA_900128675.1	Scaffold, SRA	ERR573221	2010	France	ST2250	-	-	-	5
PRJEB1915	SAMEA2662140	2588STDY5890629	*Staphylococcus argenteus*	GCA_900128635.1	Scaffold, SRA	ERR715701	2009	Singapore	ST2250	-	-	-	5
PRJEB1915	SAMEA2661974	2588STDY5890661	*Staphylococcus argenteus*	GCA_900128625.1	Scaffold, SRA	ERR715732	2011	Singapore	ST2250	-	-	-	5
PRJEB1915	SAMEA2662072	2588STDY5890701	*Staphylococcus argenteus*	GCA_900128655.1	Contig, SRA	ERR708411	2011	Malaysia	ST2250	-	-	-	5
PRJEB1915	SAMEA2662088	2588STDY5890708	*Staphylococcus argenteus*	GCA_900128645.1	Scaffold, SRA	ERR708416	2011	Malaysia	ST2250	-	-	-	5
PRJEB1915	SAMEA2662156	2588STDY5890732	*Staphylococcus argenteus*	GCA_900128705.1	Scaffold, SRA	ERR708435	2011	Malaysia	ST2250	-	-	-	5
PRJEB1915	SAMEA2662160	2588STDY5890733	*Staphylococcus argenteus*	GCA_900128615.1	Scaffold, SRA	ERR708436	2011	Malaysia	ST2250	-	-	-	5
PRJEB1915	SAMEA2662127	2588STDY5890798	*Staphylococcus argenteus*	GCA_900128665.1	Scaffold, SRA	ERR708323	2009	Israel	ST2250	-	-	-	5
PRJEB1915	SAMEA2662162	2588STDY5890811	*Staphylococcus argenteus*	GCA_900128685.1	Scaffold, SRA	ERR731038	2011	Thailand	ST2250	-	-	-	5
PRJEB1915	SAMEA2662291	2588STDY5890910	*Staphylococcus argenteus*	GCA_900128715.1	Scaffold, SRA	ERR708366	2011	Malaysia	ST1223	-	-	-	5
PRJEB1915	SAMEA2662315	2588STDY5890934	*Staphylococcus argenteus*	GCA_900128695.1	Scaffold, SRA	ERR708374	2011	Malaysia	ST2198	-	-	-	5
PRJEB1915	SAMEA2710365	2588STDY5962135	*Staphylococcus argenteus*	GCA_900128605.1	Scaffold, SRA	ERR743022	2011	Thailand	ST2250	-	-	-	5
PRJEB20633	SAMEA104034176	D7903	*Staphylococcus argenteus*	GCA_900183905.1	Contig	-	2013	Denmark	ST1223	+	-	Soft-skin infection or carrier	5
PRJEB20633	SAMEA104034164	H1540	*Staphylococcus argenteus*	GCA_900183815.1	Scaffold	-	2014	Denmark	ST2250	+	-	Soft-skin infection or carrier	5
PRJEB20633	SAMEA104034165	H1604	*Staphylococcus argenteus*	GCA_900183775.1	Scaffold	-	2014	Denmark	ST2250	+	-	Soft-skin infection or carrier	5
PRJEB20633	SAMEA104034166	H1826	*Staphylococcus argenteus*	GCA_900183825.1	Scaffold	-	2014	Denmark	ST2250	+	-	Soft-skin infection or carrier	5
PRJEB20633	SAMEA104034167	H1864	*Staphylococcus argenteus*	GCA_900183745.1	Scaffold	-	2013	Denmark	ST2250	+	-	Soft-skin infection or carrier	5
PRJEB20633	SAMEA104034172	H1955	*Staphylococcus argenteus*	GCA_900183725.1	Scaffold	-	2013	Denmark	ST2793	+	-	Soft-skin infection or carrier	5
PRJEB20633	SAMEA104034175	H2179	*Staphylococcus argenteus*	GCA_900183785.1	Contig	-	2013	Denmark	ST2250	+	-	Soft-skin infection or carrier	5
PRJEB20633	SAMEA104034155	M3040	*Staphylococcus argenteus*	GCA_900183955.1	Scaffold	-	2016	Denmark	ST2250	+	-	Soft-skin infection or carrier	5
PRJEB20633	SAMEA104034168	M4143	*Staphylococcus argenteus*	GCA_900183925.1	Scaffold	-	2013	Denmark	ST2250	+	-	Soft-skin infection or carrier	5
PRJEB20633	SAMEA104034169	M4146	*Staphylococcus argenteus*	GCA_900183755.1	Scaffold	-	2013	Denmark	ST2250	+	-	Soft-skin infection or carrier	5
PRJEB20633	SAMEA104034170	M4148	*Staphylococcus argenteus*	GCA_900183875.1	Scaffold	-	2013	Denmark	ST2250	+	-	Soft-skin infection or carrier	5
PRJEB20633	SAMEA104034171	M4185	*Staphylococcus argenteus*	GCA_900183735.1	Scaffold	-	2013	Denmark	ST1223	+	-	Soft-skin infection or carrier	5
PRJEB20633	SAMEA104034173	M4528	*Staphylococcus argenteus*	GCA_900183885.1	Contig	-	2013	Denmark	ST1223	+	-	Soft-skin infection or carrier	5
PRJEB20633	SAMEA104034174	M4611	*Staphylococcus argenteus*	GCA_900183845.1	Contig	-	2013	Denmark	ST2250	+	-	Soft-skin infection or carrier	5
PRJEB20633	SAMEA104034178	M5200	*Staphylococcus argenteus*	GCA_900183835.1	Contig	-	2016	Denmark	ST2793	+	-	Soft-skin infection or carrier	5
PRJEB20633	SAMEA104034177	M5219	*Staphylococcus argenteus*	GCA_900183915.1	Contig	-	2016	Denmark	ST2793	+	-	Soft-skin infection or carrier	5
PRJEB20633	SAMEA104034179	M5224	*Staphylococcus argenteus*	GCA_900183935.1	Contig	-	2016	Denmark	ST2793	+	-	Soft-skin infection or carrier	5
PRJEB20633	SAMEA104034156	O-1	*Staphylococcus argenteus*	GCA_900183865.1	Scaffold	-	2016	Denmark	ST2250	-	-	Soft-skin infection or carrier	5
PRJEB20633	SAMEA104034163	O-10	*Staphylococcus argenteus*	GCA_900183855.1	Scaffold	-	2015	Denmark	ST2250	-	-	Soft-skin infection or carrier	5
PRJEB20633	SAMEA104034157	O-2	*Staphylococcus argenteus*	GCA_900183965.1	Scaffold	-	2016	Denmark	unkown	-	-	Soft-skin infection or carrier	5
PRJEB20633	SAMEA104034158	O-3	*Staphylococcus argenteus*	GCA_900183765.1	Scaffold	-	2016	Denmark	ST2250	-	-	Soft-skin infection or carrier	5
PRJEB20633	SAMEA104034159	O-4	*Staphylococcus argenteus*	GCA_900183795.1	Scaffold	-	2015	Denmark	ST2250	-	-	Soft-skin infection or carrier	5
PRJEB20633	SAMEA104034160	O-5	*Staphylococcus argenteus*	GCA_900183895.1	Scaffold	-	2015	Denmark	ST2250	-	-	Soft-skin infection or carrier	5
PRJEB20633	SAMEA104034161	O-6	*Staphylococcus argenteus*	GCA_900183945.1	Scaffold	-	2015	Denmark	ST2250	-	-	Soft-skin infection or carrier	5
PRJEB20633	SAMEA104034162	O-9	*Staphylococcus argenteus*	GCA_900183805.1	Scaffold	-	2015	Denmark	ST2854	-	-	Soft-skin infection or carrier	5
PRJEB29738	SAMEA5936691	G18754525	*Staphylococcus argenteus*	-	SRA	ERR4781771	-	UK	ST2250	+	-	-	
PRJEB29738	SAMEA5936686	G18754612	*Staphylococcus argenteus*	-	SRA	ERR4791732	-	UK	ST2250	-	-	-	
PRJEB29738	SAMEA5759303	G18754695	*Staphylococcus argenteus*	-	SRA	ERR4635572	-	UK	ST2250	+	-	-	
PRJEB29738	SAMEA5759310	G18754745	*Staphylococcus argenteus*	-	SRA	ERR4661711	-	UK	ST2250	+	-	-	
PRJEB29738	SAMEA5759313	G18754754	*Staphylococcus argenteus*	-	SRA	ERR4661714	-	UK	ST2793	+	-	-	
PRJEB29738	SAMEA5759165	G18760340	*Staphylococcus argenteus*	-	SRA	ERR4766696	-	UK	ST2250	+	-	-	
PRJEB29738	SAMEA5759184	G18760797	*Staphylococcus argenteus*	-	SRA	ERR4635498	-	UK	ST2793	+	-	-	
PRJEB29738	SAMEA5759212	G18761989	*Staphylococcus argenteus*	-	SRA	ERR4635523	-	UK	ST2793	+	-	-	
PRJEB29738	SAMEA5759227	G18762363	*Staphylococcus argenteus*	-	SRA	ERR4635534	-	UK	ST2793	+	-	-	
PRJEB29738	SAMEA5759228	G18762364	*Staphylococcus argenteus*	-	SRA	ERR4635535	-	UK	ST2793	+	-	-	
PRJEB29738	SAMEA5759241	G18762634	*Staphylococcus argenteus*	-	SRA	ERR4661812	-	UK	ST2793	+	-	-	
PRJEB29738	SAMEA5936669	G18763441	*Staphylococcus argenteus*	-	SRA	ERR4791725	-	UK	ST2793	+	-	-	

PRJEB33885	SAMEA5852053	MGYG-HGUT-02548	*Staphylococcus argenteus*	GCA_902388265.1	Contig	-	-	China	ST2250	-	-	human gut	
PRJEB33933	SAMEA5858285	RIVM02149_Sar	*Staphylococcus argenteus*	-	SRA	ERR3473745	-	Netherlands	ST2250	+	-	-	
PRJEB34189	SAMEA5923362	MUMC_argenteus_1	*Staphylococcus argenteus*	-	SRA	ERR3501086	2018	Netherlands	ST1850	+	-	-	
PRJEB36681	SAMEA6530084	T151204	*Staphylococcus argenteus*	-	SRA	ERR3890992	-	Denmark	ST2250	-	prosthetic hip joint infection	blood culture	6
PRJEB37711	SAMEA6657722	1D-1445	*Staphylococcus argenteus*	-	SRA	ERR4014780	2018	Denmark	ST2250	-	-	Swab wound	
PRJEB37711	SAMEA6658173	1D-1902	*Staphylococcus argenteus*	-	SRA	ERR4015230	2018	Denmark	ST2250	-	-	Swab wound	
PRJEB37711	SAMEA6656620	1D-304	*Staphylococcus argenteus*	-	SRA	ERR4013680	2018	Denmark	ST2250	-	-	Pus	
PRJEB6386	SAMEA2039165	M260_MSHR	*Staphylococcus argenteus*	GCA_000751995.1	Contig, SRA	ERR178742	-	-	ST2198	-	-	-	
PRJEB6387	SAMEA2007996	FJABA32044V6S1	*Staphylococcus argenteus*	GCA_000751815.1	Scaffold, SRA	ERR178639	-	-	unkown	-	-	-	7
PRJEB6393	SAMEA2007935	LBSA043	*Staphylococcus argenteus*	GCA_000752055.1	Contig, SRA	ERR178673	-	-	ST2250	-	-	-	7
PRJEB6394	SAMEA2007998	M051_MSHR	*Staphylococcus argenteus*	GCA_000751495.1	Contig, SRA	ERR178693	-	-	ST1223	-	-	-	7
PRJEB6396	SAMEA1557135	H115100079	*Staphylococcus argenteus*	GCA_000752015.1	Contig, SRA	ERR182547	-	-	ST2793	+	-	-	7
PRJEB6403	SAMEA3724094	NCTC13711	*Staphylococcus argenteus*	GCA_900457475.1	Contig, SRA	ERR1351127	2006	Australia	ST1850	+		Blood Culture	
PRJEB8900	SAMEA3360374	SH3	*Staphylococcus argenteus*	-	SRA	ERR868121	-	China	ST2250	-	-	-	8
PRJEB9575	SAMEA3448927	3688STDY6124917	*Staphylococcus argenteus*	GCA_900128025.1	Contig, SRA	ERR1070014	2015	Thailand	ST2198	-	-	-	9
PRJEB9575	SAMEA3448988	3688STDY6124978	*Staphylococcus argenteus*	GCA_900127675.1	Scaffold, SRA	ERR1069841	2015	Thailand	ST2250	-	-	-	9
PRJEB9575	SAMEA3449070	3688STDY6125062	*Staphylococcus argenteus*	GCA_900128145.1	Contig, SRA	ERR1070070	2015	Thailand	ST2250	-	-	-	9
PRJEB9575	SAMEA3449071	3688STDY6125063	*Staphylococcus argenteus*	GCA_900126525.1	Contig, SRA	ERR1070071	2015	Thailand	ST2250	-	-	-	9
PRJEB9575	SAMEA3449072	3688STDY6125064	*Staphylococcus argenteus*	GCA_900126555.1	Contig, SRA	ERR1070072	2015	Thailand	ST2250	-	-	-	9
PRJEB9575	SAMEA3449073	3688STDY6125065	*Staphylococcus argenteus*	GCA_900126545.1	Contig, SRA	ERR1070073	2015	Thailand	ST2250	-	-	-	9
PRJEB9575	SAMEA3449074	3688STDY6125066	*Staphylococcus argenteus*	GCA_900126565.1	Contig, SRA	ERR1070074	2015	Thailand	ST2250	-	-	-	9
PRJEB9575	SAMEA3449075	3688STDY6125067	*Staphylococcus argenteus*	GCA_900128155.1	Contig, SRA	ERR1070075	2015	Thailand	ST2250	-	-	-	9
PRJEB9575	SAMEA3449076	3688STDY6125068	*Staphylococcus argenteus*	GCA_900128165.1	Contig, SRA	ERR1070076	2015	Thailand	ST2250	-	-	-	9
PRJEB9575	SAMEA3449077	3688STDY6125069	*Staphylococcus argenteus*	GCA_900128175.1	Contig, SRA	ERR1070077	2015	Thailand	ST2250	-	-	-	9
PRJEB9575	SAMEA3449078	3688STDY6125070	*Staphylococcus argenteus*	GCA_900126585.1	Contig, SRA	ERR1070078	2015	Thailand	ST2250	-	-	-	9
PRJEB9575	SAMEA3449079	3688STDY6125071	*Staphylococcus argenteus*	GCA_900126595.1	Contig, SRA	ERR1070079	2015	Thailand	ST2250	-	-	-	9
PRJEB9575	SAMEA3449080	3688STDY6125072	*Staphylococcus argenteus*	GCA_900126605.1	Contig, SRA	ERR1070080	2015	Thailand	ST2250	-	-	-	9
PRJEB9575	SAMEA3449081	3688STDY6125073	*Staphylococcus argenteus*	GCA_900128185.1	Contig, SRA	ERR1070081	2015	Thailand	ST2250	-	-	-	9
PRJEB9575	SAMEA3449082	3688STDY6125074	*Staphylococcus argenteus*	GCA_900126615.1	Scaffold, SRA	ERR1070082	2015	Thailand	ST2250	-	-	-	9
PRJEB9575	SAMEA3449083	3688STDY6125075	*Staphylococcus argenteus*	GCA_900128195.1	Contig, SRA	ERR1070083	2015	Thailand	ST2250	-	-	-	9
PRJEB9575	SAMEA3449084	3688STDY6125076	*Staphylococcus argenteus*	GCA_900126625.1	Contig, SRA	ERR1070084	2015	Thailand	ST2250	-	-	-	9
PRJEB9575	SAMEA3449085	3688STDY6125077	*Staphylococcus argenteus*	GCA_900128205.1	Contig, SRA	ERR1070085	2015	Thailand	ST2250	-	-	-	9
PRJEB9575	SAMEA3449086	3688STDY6125078	*Staphylococcus argenteus*	GCA_900126645.1	Scaffold, SRA	ERR1070086	2015	Thailand	ST2250	-	-	-	9
PRJEB9575	SAMEA3449087	3688STDY6125079	*Staphylococcus argenteus*	GCA_900126655.1	Contig, SRA	ERR1070087	2015	Thailand	ST2250	-	-	-	9
PRJEB9575	SAMEA3449088	3688STDY6125080	*Staphylococcus argenteus*	GCA_900126665.1	Contig, SRA	ERR1070088	2015	Thailand	ST2250	-	-	-	9
PRJEB9575	SAMEA3449089	3688STDY6125081	*Staphylococcus argenteus*	GCA_900126675.1	Scaffold, SRA	ERR1070089	2015	Thailand	ST2250	-	-	-	9
PRJEB9575	SAMEA3449090	3688STDY6125082	*Staphylococcus argenteus*	GCA_900128215.1	Scaffold, SRA	ERR1070090	2015	Thailand	ST2250	-	-	-	9
PRJEB9575	SAMEA3449091	3688STDY6125083	*Staphylococcus argenteus*	GCA_900126685.1	Contig, SRA	ERR1070091	2015	Thailand	ST2250	-	-	-	9
PRJEB9575	SAMEA3449092	3688STDY6125084	*Staphylococcus argenteus*	GCA_900126695.1	Scaffold, SRA	ERR1070092	2015	Thailand	ST2250	-	-	-	9
PRJEB9575	SAMEA3449093	3688STDY6125085	*Staphylococcus argenteus*	GCA_900128225.1	Contig, SRA	ERR1070093	2015	Thailand	ST2250	-	-	-	9
PRJEB9575	SAMEA3449094	3688STDY6125086	*Staphylococcus argenteus*	GCA_900128235.1	Contig, SRA	ERR1070094	2015	Thailand	ST2250	-	-	-	9
PRJEB9575	SAMEA3449095	3688STDY6125087	*Staphylococcus argenteus*	GCA_900126705.1	Contig, SRA	ERR1070095	2015	Thailand	ST2250	-	-	-	9
PRJEB9575	SAMEA3449096	3688STDY6125088	*Staphylococcus argenteus*	GCA_900128245.1	Contig, SRA	ERR1070096	2015	Thailand	ST2250	-	-	-	9
PRJEB9575	SAMEA3449097	3688STDY6125089	*Staphylococcus argenteus*	GCA_900128255.1	Contig, SRA	ERR1070097	2015	Thailand	ST2250	-	-	-	9
PRJEB9575	SAMEA3449098	3688STDY6125090	*Staphylococcus argenteus*	GCA_900126725.1	Contig, SRA	ERR1070098	2015	Thailand	ST2250	-	-	-	9
PRJEB9575	SAMEA3449099	3688STDY6125091	*Staphylococcus argenteus*	GCA_900128265.1	Contig, SRA	ERR1070099	2015	Thailand	ST2250	-	-	-	9
PRJEB9575	SAMEA3449100	3688STDY6125092	*Staphylococcus argenteus*	GCA_900128275.1	Contig, SRA	ERR1070100	2015	Thailand	unkown	-	-	-	9
PRJEB9575	SAMEA3449101	3688STDY6125093	*Staphylococcus argenteus*	GCA_900126735.1	Contig, SRA	ERR1070101	2015	Thailand	ST2250	-	-	-	9
PRJEB9575	SAMEA3449102	3688STDY6125100	*Staphylococcus argenteus*	GCA_900126745.1	Contig, SRA	ERR1070102	2015	Thailand	ST2250	-	-	-	9
PRJEB9575	SAMEA3449107	3688STDY6125105	*Staphylococcus argenteus*	GCA_900126775.1	Contig, SRA	ERR1070107	2015	Thailand	ST2250	-	-	-	9
PRJEB9575	SAMEA3449108	3688STDY6125106	*Staphylococcus argenteus*	GCA_900126785.1	Contig, SRA	ERR1070108	2015	Thailand	ST2250	-	-	-	9
PRJEB9575	SAMEA3449110	3688STDY6125108	*Staphylococcus argenteus*	GCA_900126795.1	Contig, SRA	ERR1070110	2015	Thailand	ST2250	-	-	-	9
PRJEB9575	SAMEA3449111	3688STDY6125109	*Staphylococcus argenteus*	GCA_900126805.1	Scaffold, SRA	ERR1070111	2015	Thailand	ST2250	-	-	-	9
PRJEB9575	SAMEA3449112	3688STDY6125110	*Staphylococcus argenteus*	GCA_900126815.1	Contig, SRA	ERR1070112	2015	Thailand	ST2250	-	-	-	9
PRJEB9575	SAMEA3449113	3688STDY6125111	*Staphylococcus argenteus*	GCA_900126825.1	Contig, SRA	ERR1070113	2015	Thailand	ST2250	-	-	-	9
PRJEB9575	SAMEA3449114	3688STDY6125112	*Staphylococcus argenteus*	GCA_900126835.1	Scaffold, SRA	ERR1070114	2015	Thailand	ST2250	-	-	-	9
PRJEB9575	SAMEA3449115	3688STDY6125113	*Staphylococcus argenteus*	GCA_900128325.1	Contig, SRA	ERR1070115	2015	Thailand	ST2250	-	-	-	9
PRJEB9575	SAMEA3449116	3688STDY6125114	*Staphylococcus argenteus*	GCA_900128335.1	Contig, SRA	ERR1070116	2015	Thailand	ST2250	-	-	-	9
PRJEB9575	SAMEA3449117	3688STDY6125115	*Staphylococcus argenteus*	GCA_900126855.1	Contig, SRA	ERR1070117	2015	Thailand	ST2250	-	-	-	9
PRJEB9575	SAMEA3449118	3688STDY6125116	*Staphylococcus argenteus*	GCA_900126875.1	Scaffold, SRA	ERR1070118	2015	Thailand	ST2250	-	-	-	9
PRJEB9575	SAMEA3449119	3688STDY6125117	*Staphylococcus argenteus*	GCA_900126885.1	Scaffold, SRA	ERR1070119	2015	Thailand	ST2250	-	-	-	9
PRJEB9575	SAMEA3449120	3688STDY6125118	*Staphylococcus argenteus*	GCA_900126865.1	Contig, SRA	ERR1070120	2015	Thailand	ST2250	-	-	-	9
PRJEB9575	SAMEA3449121	3688STDY6125119	*Staphylococcus argenteus*	GCA_900126895.1	Contig, SRA	ERR1070121	2015	Thailand	ST2250	-	-	-	9

PRJEB9575	SAMEA3449122	3688STDY6125120	*Staphylococcus argenteus*	GCA_900126905.1	Contig, SRA	ERR1070122	2015	Thailand	ST2250	-	-	-	9
PRJEB9575	SAMEA3449123	3688STDY6125121	*Staphylococcus argenteus*	GCA_900126915.1	Contig, SRA	ERR1070123	2015	Thailand	ST2250	-	-	-	9
PRJEB9575	SAMEA3449124	3688STDY6125122	*Staphylococcus argenteus*	GCA_900128345.1	Contig, SRA	ERR1070124	2015	Thailand	ST2250	-	-	-	9
PRJEB9575	SAMEA3449125	3688STDY6125123	*Staphylococcus argenteus*	GCA_900128355.1	Contig, SRA	ERR1070125	2015	Thailand	ST2250	-	-	-	9
PRJEB9575	SAMEA3449126	3688STDY6125124	*Staphylococcus argenteus*	GCA_900128365.1	Scaffold, SRA	ERR1070126	2015	Thailand	ST2250	-	-	-	9
PRJEB9575	SAMEA3449127	3688STDY6125125	*Staphylococcus argenteus*	GCA_900126935.1	Scaffold, SRA	ERR1070127	2015	Thailand	ST2250	-	-	-	9
PRJEB9575	SAMEA3449128	3688STDY6125126	*Staphylococcus argenteus*	GCA_900126945.1	Scaffold, SRA	ERR1070128	2015	Thailand	ST2250	-	-	-	9
PRJEB9575	SAMEA3449129	3688STDY6125127	*Staphylococcus argenteus*	GCA_900126955.1	Contig, SRA	ERR1070129	2015	Thailand	ST2198	-	-	-	9
PRJEB9575	SAMEA3449130	3688STDY6125128	*Staphylococcus argenteus*	GCA_900126965.1	Contig, SRA	ERR1070130	2015	Thailand	ST1223	-	-	-	9
PRJEB9575	SAMEA3449131	3688STDY6125129	*Staphylococcus argenteus*	GCA_900128385.1	Contig, SRA	ERR1070131	2015	Thailand	ST2854	-	-	-	9
PRJEB9575	SAMEA3449132	3688STDY6125130	*Staphylococcus argenteus*	GCA_900126975.1	Contig, SRA	ERR1070132	2015	Thailand	ST2854	-	-	-	9
PRJEB9575	SAMEA3449133	3688STDY6125131	*Staphylococcus argenteus*	GCA_900126985.1	Contig, SRA	ERR1070133	2015	Thailand	ST2250	-	-	-	9
PRJEB9575	SAMEA3449134	3688STDY6125132	*Staphylococcus argenteus*	GCA_900126995.1	Contig, SRA	ERR1070134	2015	Thailand	ST1223	-	-	-	9
PRJEB9575	SAMEA3449135	3688STDY6125133	*Staphylococcus argenteus*	GCA_900127005.1	Scaffold, SRA	ERR1070135	2015	Thailand	ST1223	-	-	-	9
PRJEB9575	SAMEA3449136	3688STDY6125134	*Staphylococcus argenteus*	GCA_900127025.1	Scaffold, SRA	ERR1070136	2015	Thailand	ST2854	-	-	-	9
PRJEB9575	SAMEA3449137	3688STDY6125135	*Staphylococcus argenteus*	GCA_900127035.1	Contig, SRA	ERR1070137	2015	Thailand	ST1223	-	-	-	9
PRJEB9575	SAMEA3449138	3688STDY6125136	*Staphylococcus argenteus*	GCA_900127045.1	Contig, SRA	ERR1070138	2015	Thailand	ST2250	-	-	-	9
PRJEB9575	SAMEA3449139	3688STDY6125137	*Staphylococcus argenteus*	GCA_900127055.1	Contig, SRA	ERR1070139	2015	Thailand	ST2198	-	-	-	9
PRJEB9575	SAMEA3449140	3688STDY6125138	*Staphylococcus argenteus*	GCA_900127075.1	Contig, SRA	ERR1070140	2015	Thailand	ST2250	-	-	-	9
PRJEB9575	SAMEA3449141	3688STDY6125139	*Staphylococcus argenteus*	GCA_900127065.1	Contig, SRA	ERR1070141	2015	Thailand	ST1223	-	-	-	9
PRJEB9575	SAMEA3449142	3688STDY6125140	*Staphylococcus argenteus*	GCA_900127085.1	Contig, SRA	ERR1070142	2015	Thailand	ST2250	-	-	-	9
PRJEB9575	SAMEA3449145	3688STDY6125143	*Staphylococcus argenteus*	GCA_900127115.1	Contig, SRA	ERR1070145	2015	Thailand	ST1223	-	-	-	9
PRJNA185403	SAMN03817247	PR02	*Staphylococcus argenteus*	GCA_003569945.1	Scaffold	-	2009	Malaysia	ST2854	-	septicemia	blood from patient with septicemia	
PRJNA227677	SAMN02402601	F87619	*Staphylococcus argenteus*	GCA_000602805.1	Contig, SRA	SRR1165388, SRR1167068	2013-01-18	-	ST2250	+	-	-	10
PRJNA227695	SAMN02402619	M21126	*Staphylococcus argenteus*	GCA_000606605.1	Scaffold, SRA	SRR1165455, SRR1167129	2013-04-08	-	ST2250	+	-	-	10
PRJNA267549	SAMN03197271	1299_SAUR	*Staphylococcus argenteus*	GCA_001061465.1	Contig, SRA	SRR1655503	Jul-16	USA:WA	ST2250	-	-	-	10
PRJNA269675	SAMN08689422	SCPM-O-B-8378	*Staphylococcus argenteus*	GCA_003011845.1	Scaffold	SRR6828694	Mar-2015	-	ST2250	-	-	-	
PRJNA302939	SAMN04286974	ABFQM	*Staphylococcus argenteus*	GCA_002221825.1	Contig, SRA	SRR3574737, SRR3574739	2015	USA	ST2198	-	-	-	
PRJNA305687	SAMN07566689	CCUG69384	*Staphylococcus argenteus*	GCA_003010635.1	Contig	-	16-Aug-2016	Sweden	ST2250	+	-	Perineum	11
PRJNA305687	SAMN07566687	CCUG69385	*Staphylococcus argenteus*	GCA_003022395.1	Contig	-	11-Aug-2016	Sweden	ST1223	+	-	throat	11
PRJNA310972	SAMN04457463	RK308	*Staphylococcus argenteus*	GCA_001641625.1	Contig	-	Mar-2015	Sweden	ST2250	+	-	abscess	12
PRJNA317277	SAMN04604742	SJTU-F20124	*Staphylococcus argenteus*	GCA_001969635.1	Scaffold	SRR3329440	2005	China	ST3261	-	-	-	10
PRJNA317278	SAMN04604743	SJTU-F20419	*Staphylococcus argenteus*	GCA_001969695.1	Contig	SRR3329633	2012	China	ST2250	-	-	-	10
PRJNA317279	SAMN04604744	SJTU-F21164	*Staphylococcus argenteus*	GCA_001969705.1	Scaffold	SRR3329803	2011	China	ST2250	-	-	-	10
PRJNA317280	SAMN04604745	SJTU-F21224	*Staphylococcus argenteus*	GCA_001969715.1	Contig	SRR3329804	2012	China	ST2250	-	-	-	10
PRJNA317282	SAMN04604746	SJTU-F21285	*Staphylococcus argenteus*	GCA_001969725.1	Contig	SRR3329805	2012	China	ST2250	-	-	pork	10
PRJNA321471	SAMN04994883	BN75	*Staphylococcus argenteus*	GCA_001891145.1	Chromosome	-	11-Apr-2012	Gabon	ST2198	-	-	feces of Gorilla	13
PRJNA339206	SAMN06177165	DSM28299	*Staphylococcus argenteus*	GCA_002901785.1	Scaffold, SRA	SRR5127999	2006	Australia	ST1850	+	-	blood	
PRJNA398564	SAMN07510741	XNO62	*Staphylococcus argenteus*	GCA_003595345.1	Complete	-	2014-09-29	China	ST2250	-	prosthetic joint infection	joint fluid	14
PRJNA400109	SAMN07510742	XNO106	*Staphylococcus argenteus*	GCA_002812345.2	Complete	-	2015-01-05	China	ST2250	-	-	-	14
PRJNA434395	SAMN08556530	PH3432	*Staphylococcus argenteus*	GCA_005472255.1	Contig	-	23-Oct-2017	Canada	ST2250	+	-	Nose	
PRJNA476500	SAMN09435802	ST2250	*Staphylococcus argenteus*	GCA_003935695.1	Contig	SRR8282609	2012	China	ST2250	-	-	pig	
PRJNA482405	SAMN09704415	PHL3431	*Staphylococcus argenteus*	GCA_009874245.1	Contig	-	Oct-2017	Canada	ST2250	+	-	Rectum	
PRJNA482411	SAMN09704514	PHL3433	*Staphylococcus argenteus*	GCA_008368955.1	Contig	-	Oct-2017	Canada	ST2250	+	-	nares	
PRJNA530346	SAMN11311880	Arg23	*Staphylococcus argenteus*	-	SRA	SRR8835651	2008	France	unkown	-	chronic prothetic joint infection	blood culture	15
PRJNA530346	SAMN11311881	ArgPVL	*Staphylococcus argenteus*	-	SRA	SRR8835652	2014	France	ST2250	-	sepsis	blood culture	15
PRJNA555633	SAMN12340634	B3-25B	*Staphylococcus argenteus*	GCA_007859575.1	Complete, SRA	SRR9943105	2010-06	USA	ST2198	-	-	retail beef liver	16
PRJNA595347	SAMN13979052	5506	*Staphylococcus argenteus*	GCA_010570875.1	Contig	-	2018	Colombia	ST2250	-	-	blood	
PRJNA633996	SAMN15184454	18A183	*Staphylococcus argenteus*	-	SRA	SRR12472757	2018	China	ST1223	+	-	food	
PRJNA633996	SAMN15184474	CFSA15SA062	*Staphylococcus argenteus*	-	SRA	SRR12472735	2015	China	ST2250	-	-	food	
PRJNA633996	SAMN15184478	CFSA17SA018	*Staphylococcus argenteus*	-	SRA	SRR12472730	2017	China	ST1223	-	-	food	
PRJNA633996	SAMN15184479	CFSA17SA053	*Staphylococcus argenteus*	-	SRA	SRR12472729	2017	China	ST1223	-	-	food	
PRJNA633996	SAMN15184480	CFSA17SA054	*Staphylococcus argenteus*	-	SRA	SRR12472728	2017	China	ST1223	-	-	food	
PRJNA641487	SAMN15354747	058S_SR	*Staphylococcus argenteus*	-	SRA	SRR12078850	2018	Samoa	ST2250	-	-	-	
PRJNA641487	SAMN15354748	117N_SR	*Staphylococcus argenteus*	-	SRA	SRR12078849	2018	Samoa	ST2250	-	-	-	
PRJNA641487	SAMN15354749	305N_SR	*Staphylococcus argenteus*	-	SRA	SRR12078848	2018	Samoa	unkown	-	-	-	
PRJNA641487	SAMN15354750	752S_SR	*Staphylococcus argenteus*	-	SRA	SRR12078847	2018	Samoa	ST2250	-	-	-	
PRJNA646358	SAMN15541329	BAL1928	*Staphylococcus argenteus*	-	SRA	SRR12233541	Nov-2014	Vietnam	ST2250	-	Colonization	endotracheal aspirate	
PRJNA646358	SAMN15541330	BAL1935	*Staphylococcus argenteus*	-	SRA	SRR12233530	Nov-2014	Vietnam	ST2250	-	Colonization	endotracheal aspirate	
PRJNA646358	SAMN15541331	BAL1945	*Staphylococcus argenteus*	-	SRA	SRR12233519	Nov-2014	Vietnam	ST2250	-	pneumonia	endotracheal aspirate	
PRJNA646358	SAMN15541339	BAL2160	*Staphylococcus argenteus*	-	SRA	SRR12233453	Nov-2014	Vietnam	ST1223	+	pneumonia	endotracheal aspirate	
PRJNA646358	SAMN15541344	BC15-02467	*Staphylococcus argenteus*	-	SRA	SRR12233448	Nov-2014	Vietnam	ST2250	-	Bloodstream infection	blood culture	
PRJNA646358	SAMN15541358	NS2714	*Staphylococcus argenteus*	-	SRA	SRR12233432	Nov-2014	Vietnam	ST2250	-	Colonization	nasal swab	

PRJNA646358	SAMN15541367	NS3791	*Staphylococcus argenteus*	-	SRA	SRR12233422	Nov-2014	Vietnam	ST1223	+	Colonization	nasal swab	
PRJNA646358	SAMN15541368	NS425	*Staphylococcus argenteus*	-	SRA	SRR12233421	Nov-2014	Vietnam	ST2250	-	Colonization	nasal swab	
PRJNA666697	SAMN16304017	DSM28299	*Staphylococcus argenteus*	GCA_014877305.1	Contig	-	2006	Australia	ST1850	+	-	blood	
PRJNA666697	SAMN16304019	MC2	*Staphylococcus argenteus*	GCA_014877005.1	Contig	-	2019	USA	ST2250	-	-	urine	
PRJNA666697	SAMN16304021	MC3	*Staphylococcus argenteus*	GCA_014877945.1	Contig	-	11-Mar-2019	USA	ST2250	-	-	Buttocks wound	
PRJNA666697	SAMN16304022	MC4	*Staphylococcus argenteus*	GCA_014877235.1	Contig	-	2019	USA	ST2250	-	-	blood	
PRJNA666697	SAMN16304011	PHL1144	*Staphylococcus argenteus*	GCA_014877495.1	Contig	-	26-Feb-2019	Canada	ST1223	-	-	Rectal and wound swab	
PRJNA666697	SAMN16304010	PHL2411	*Staphylococcus argenteus*	GCA_014877555.1	Contig	-	07-Mar-2019	Canada	ST2198	-	-	tendon tissue	
PRJNA666697	SAMN16304007	PHL2420	*Staphylococcus argenteus*	GCA_014878065.1	Contig	-	31-Dec-2019	Canada	ST2250	-	-	Stump Wound	
PRJNA666697	SAMN16304006	PHL3446	*Staphylococcus argenteus*	GCA_014878035.1	Contig	-	2019/1/21	Canada	ST2250	-	-	Tissue	
PRJNA666697	SAMN16304014	PHL4226	*Staphylococcus argenteus*	GCA_014877505.1	Contig	-	30-Oct-2019	Canada	ST2250	-	-	blood	
PRJNA666697	SAMN16304015	PHL4313	*Staphylococcus argenteus*	GCA_014877835.1	Contig	-	03-Nov-2019	Canada	ST1223	-	-	Synovial fluid	
PRJNA666697	SAMN16304016	PHL4553	*Staphylococcus argenteus*	GCA_014877425.1	Contig	-	09-Nov-2019	Canada	unkown	-	-	blood	
PRJNA666697	SAMN16304012	PHL4815	*Staphylococcus argenteus*	GCA_014877035.1	Contig	-	01-Mar-2019	Canada	ST2250	-	-	Head Wound	
PRJNA666697	SAMN16304005	PHL5740	*Staphylococcus argenteus*	GCA_014877595.1	Contig	-	17-Oct-2018	Canada	ST1223	-	-	Eye Wound	
PRJNA666697	SAMN16304008	PHL6318	*Staphylococcus argenteus*	GCA_014878055.1	Contig	-	07-Jan-2019	Canada	ST2250	-	-	Toe Wound	
PRJNA666697	SAMN16304004	PHL6344	*Staphylococcus argenteus*	GCA_014878095.1	Contig	-	12-Nov-2018	Canada	ST2250	-	-	cheek	
PRJNA666697	SAMN16304009	PHL8605	*Staphylococcus argenteus*	GCA_014877525.1	Contig	-	10-Dec-2018	Canada	ST2250	-	-	sternal wound	
PRJNA666697	SAMN16304013	PHL8642	*Staphylococcus argenteus*	GCA_014877515.1	Contig	-	26-Mar-19	Canada	unkown	-	-	eye	
PRJNA666697	SAMN16304023	WU1	*Staphylococcus argenteus*	GCA_014877475.1	Contig	-	2019	USA	ST2250	-	-	Foot tissue	
PRJNA666697	SAMN16304024	WU2	*Staphylococcus argenteus*	GCA_014877415.1	Contig	-	2019	USA	ST2250	-	Buttocks wound	-	
PRJNA666697	SAMN16304020	WU3	*Staphylococcus argenteus*	GCA_014877915.1	Contig	-	2019	USA	ST2250	-	-	Deep throat swab	
PRJDB11096	SAMD00276358	JARB-RP-0005 (SG-01)	*Staphylococcus argenteus*	-	SRA	DRR270463	Mar-2019	Japan	ST2250	-	-	sputum	17
PRJDB11096	SAMD00276359	JARB-RP-0006 (SG-02)	*Staphylococcus argenteus*	-	SRA	DRR270464	Mar-2019	Japan	ST2198	-	-	urine	17
PRJDB11096	SAMD00276360	JARB-RP-0007 (SG-03)	*Staphylococcus argenteus*	-	SRA	DRR270465	Mar-2019	Japan	ST1223	-	-	dural abcess	17
PRJDB11096	SAMD00276361	JARB-RP-0008 (SG-04)	*Staphylococcus argenteus*	-	SRA	DRR270466	Apr-2019	Japan	ST2250	-	-	urine	17
PRJDB11096	SAMD00276362	JARB-RP-0009 (SG-05-1)	*Staphylococcus argenteus*	-	SRA	DRR270467	Apr-2019	Japan	ST2250	-	-	ear discharge	17
PRJDB11096	SAMD00276363	JARB-RP-0010 (SG-05-2)	*Staphylococcus argenteus*	-	SRA	DRR270468	Apr-2019	Japan	ST2250	-	-	ear discharge	17
PRJDB11096	SAMD00276364	JARB-RP-0011 (SG-06)	*Staphylococcus argenteus*	-	SRA	DRR270469	Apr-2019	Japan	ST1223	-	-	nasal ischarge	17
PRJDB11096	SAMD00276365	JARB-RP-0012 (SG-07)	*Staphylococcus argenteus*	-	SRA	DRR270470	Apr-2019	Japan	ST1223	-	-	stool	17
PRJDB11096	SAMD00276366	JARB-RP-0013 (SG-08)	*Staphylococcus argenteus*	-	SRA	DRR270471	Apr-2019	Japan	ST2198	-	-	skin	17
PRJDB11096	SAMD00276367	JARB-RP-0014 (SG-09)	*Staphylococcus argenteus*	-	SRA	DRR270472	May-2019	Japan	ST2250	-	-	nasal ischarge	17
PRJDB11096	SAMD00276368	JARB-RP-0015 (SG-10)	*Staphylococcus argenteus*	-	SRA	DRR270473	May-2019	Japan	ST2198	-	-	sputum	17
PRJDB11096	SAMD00276369	JARB-RP-0016 (SG-11)	*Staphylococcus argenteus*	-	SRA	DRR270474	May-2019	Japan	ST2250	-	-	vaginal discharge	17
PRJDB11096	SAMD00276372	JARB-RP-0019 (SG-14)	*Staphylococcus argenteus*	-	SRA	DRR270475	Jun-2019	Japan	ST2198	-	-	skin	17
PRJDB11096	SAMD00276374	JARB-RP-0021 (SG-16)	*Staphylococcus argenteus*	-	SRA	DRR270476	Jun-2019	Japan	ST2250	-	-	stool	17
PRJDB11096	SAMD00276375	JARB-RP-0022 (SG-17)	*Staphylococcus argenteus*	-	SRA	DRR270477	Jun-2019	Japan	ST1223	-	-	stool	17
PRJDB11096	SAMD00276376	JARB-RP-0023 (SG-18)	*Staphylococcus argenteus*	-	SRA	DRR270478	Jun-2019	Japan	ST1223	-	-	vaginal discharge	17
PRJDB11096	SAMD00276378	JARB-RP-0025 (SG-20)	*Staphylococcus argenteus*	-	SRA	DRR270479	Jul-2019	Japan	ST2198	-	-	pus	17
PRJDB11096	SAMD00276379	JARB-RP-0026 (SG-21)	*Staphylococcus argenteus*	-	SRA	DRR270480	Jul-2019	Japan	ST2250	-	-	sputum	17
PRJDB11096	SAMD00276380	JARB-RP-0027 (SG-22)	*Staphylococcus argenteus*	-	SRA	DRR270481	Jul-2019	Japan	ST2250	-	-	pharynx	17
PRJDB11096	SAMD00276381	JARB-RP-0028 (SG-23)	*Staphylococcus argenteus*	-	SRA	DRR270482	Jul-2019	Japan	ST2250	-	-	blood	17
PRJDB11096	SAMD00276382	JARB-RP-0029 (SG-25)	*Staphylococcus argenteus*	-	SRA	DRR270483	Jul-2019	Japan	ST2250	-	-	sputum	17
PRJDB11127	SAMD00276348	JH5333	*Staphylococcus argenteus*	-	SRA	DRR272453		Japan	ST2250	-	-	healthy nasal	This study
PRJDB11127	SAMD00276349	JH5336	*Staphylococcus argenteus*	-	SRA	DRR272454		Japan	ST1223	-	otitis	otorrhea	This study
PRJDB11127	SAMD00276350	JH5337	*Staphylococcus argenteus*	-	SRA	DRR272455		Japan	ST1223	-	otitis	otorrhea	This study
PRJDB11127	SAMD00276351	JH5338	*Staphylococcus argenteus*	-	SRA	DRR272456		Japan	ST2250	-	otitis	otorrhea	This study
PRJDB11127	SAMD00276352	JH5340	*Staphylococcus argenteus*	-	SRA	DRR272457		Japan	ST1223	-	-	healthy nasal	This study
PRJDB7256	SAMD00276347	JH6199	*Staphylococcus argenteus*	-	SRA	DRR272463		Japan	ST2250	-	cellulitis	blood culture	18
PRJDB7256	SAMD00276346	JH6211	*Staphylococcus argenteus*	-	SRA	DRR272464		Japan	ST2250	-	catheter-related blood stream infection	blood culture	18
PRJDB10984	SAMD00281814	P217	*Staphylococcus argenteus*	-	SRA	DRR276336		Japan	ST1223	-	soft skin tissue infection (openpus)	human skin	
PRJDB10984	SAMD00281815	P309	*Staphylococcus argenteus*	-	SRA	DRR276337		Japan	ST2854	-	soft skin tissue infection (open pus)	human skin	
PRJDB10984	SAMD00281816	P476	*Staphylococcus argenteus*	-	SRA	DRR276338		Japan	ST1223	-	soft skin tissue infection (non-open pus)	human skin	
PRJDB10984	SAMD00281817	P527	*Staphylococcus argenteus*	-	SRA	DRR276339		Japan	ST1223	-	soft skin tissue infection (open pus)	human skin	
PRJDB10984	SAMD00281818	P571	*Staphylococcus argenteus*	-	SRA	DRR276340		Japan	ST2854	-	soft skin tissue infection	human skin	
PRJDB10984	SAMD00281819	P729	*Staphylococcus argenteus*	-	SRA	DRR276341		Japan	ST1223	-	soft skin tissue infection (non-open pus)	human skin	
PRJDB7256	SAMD00276353	SARG1271	*Staphylococcus argenteus*	-	SRA	DRR272458		Japan	ST2250	-	atopic dermatitis	human skin (upper back)	This study
PRJDB7256	SAMD00276354	SARG1913	*Staphylococcus argenteus*	-	SRA	DRR272459		Japan	ST2250	-	atopic dermatitis	human skin (glabella)	This study
PRJDB7256	SAMD00276355	SARG2343	*Staphylococcus argenteus*	-	SRA	DRR272460		Japan	ST2250	-	atopic dermatitis	human skin (glabella)	This study
PRJDB7256	SAMD00276356	SARG3091	*Staphylococcus argenteus*	-	SRA	DRR272461		Japan	ST2250	-	atopic dermatitis	human skin (forearm palm)	This study
PRJDB7256	SAMD00276357	SARG5253	*Staphylococcus argenteus*	-	SRA	DRR272462		Japan	unkown	-	atopic dermatitis	human skin (glabella)	This study
