# Supplementary material for: Genomic analysis and identification of a novel superantigen, SargEY, in Staphylococcus argenteus isolated from atopic dermatitis lesions
Source: mSphere. 2024 Jul 11;9(7):e00505-24. doi: 10.1128/msphere.00505-24 (PMC11288046; doi:10.1128/msphere.00505-24)
Supplement: Table 3 — Primer used for toxins gene cloning. [file msphere.00505-24-s0006.pdf]

Supplementary Table 3. Primer used for toxins gene cloning.

Primer purpose	Primer name	Oligonucleotide sequence (5'- 3')	Source or reference
cloning	*sey* _argenteus_F	GGGGC**CATATG**CACCACCACCACCACCACAAAACAACTGGATTGATTACAG	This study
	sey _argenteus_R	GGGCC**GGATCC**GTTGGAACGACCTATTTC	This study
	seh _*Nco* I_ F	GCG**CCATGG**TGCACCACCACCACCACCACGAAGATTTACACGATAAAAGTG	This study
	seh _*Eco* RI_R	CCCCCC**GAATTC**TTATACTTTTTTCTTAGTATATAG	This study
	set _*Nde* I_F	GGGGC**CATATG**CACCACCACCACCACCACGATTCTCGTGAAGGTTTAAAAG	This study
	set _*Bam* HI_R	GGGCC**GGATCC**GCTCTATTTTTCCATATATATATC	This study

Enzyme restriction cut site are bold.
