# Supplementary material for: Genomic analysis and identification of a novel superantigen, SargEY, in Staphylococcus argenteus isolated from atopic dermatitis lesions
Source: mSphere. 2024 Jul 11;9(7):e00505-24. doi: 10.1128/msphere.00505-24 (PMC11288046; doi:10.1128/msphere.00505-24)
Supplement: Table 4 — Emetic activity test in monkey model. [file msphere.00505-24-s0007.pdf]

Supplementary Table 4. Emetic activity test in monkey model.

Toxin	No. of monkey vomited/tested	Latency (min)	No. of emetic episodes
$S_{arg}EY$ 250 µg/kg	2/4	167,192	3, 3
PBS	0/4		
