# Supplementary material for: Genomic analysis and identification of a novel superantigen, SargEY, in Staphylococcus argenteus isolated from atopic dermatitis lesions
Source: mSphere. 2024 Jul 11;9(7):e00505-24. doi: 10.1128/msphere.00505-24 (PMC11288046; doi:10.1128/msphere.00505-24)
Supplement: Table 5 — Recognition motifs of HsdS or HsdM in the type I-IV restriction-modification system of the S. argenteus isolates used in this study. [file msphere.00505-24-s0008.pdf]

Supplementary Table 5. Recognition motifs of HsdS or HsdM in the type I-IV restriction-modification system of the *S. argenteus* isolates used in this study.

		Restriction-Modification System[a]											
		Type I									Type II	Type III	Type IV
Strain	ST	*hsdS*[b], %Identitiy (CAAGNNNNNNTAG)	*hsdS*, %Identity (GAAGNNNNNNTGG)	*hsdM*, %Identitiy (CAAGNNNNNNTAG)	*hsdM*, %Identity (GAAGNNNNNNTGG)	*hsdS*, %Identity (CAAYNNNNNCTTC)	*hsdS*, %Identity (CAAGNNNNNRTC)	*hsdS*, %Identity (CNGANNNNNNNTTYG)	*hsdM*, %Identity (CAAGNNNNNRTC)	*hsdM*, %Identity (CNGANNNNNNNTTYG)	*hsdM*, %Identity (CCWGG)	*hsdM*, %Identity (GCTGA)	*SauNewI*, %Identity (SCNGS)
JARB-HU0072 (P102)	ST2198	-[c]	-	-	-	-	-	-	-	●, 96.3%	-	●, 99.9%	●, 98.0%
JARB-HU0252 (P396)	ST2250	●, 100%	●, 100%	●, 100%	●, 100%	-	-	-	-	-	-	-	●, 97.9%
Tokyo13064	ST1223	-	-	●, 96.4%	-	●, 95.9%	-	-	●, 96.5%	-	-	-	●, 97.9%
Tokyo13069	ST1223	-	-	●, 96.4%	-	●, 95.9%	-	-	●, 96.5%	-	-	-	●, 97.9%
58113	ST2250	●, 100%	●, 100%	●, 100%	●, 100%	-	-	-	-	-	-	-	●, 97.9%
SARG0275	ST2250	●, 100%	●, 100%	●, 100%	●, 100%	-	-	-	-	-	-	-	●, 97.9%
TWCC58113	ST2250	●, 100%	●, 100%	●, 100%	●, 100%	-	-	-	-	-	-	-	●, 97.9%
TUM19485	ST1223	-	-	●, 96.4%	-	●, 95.9%	-	-	●, 96.5%	-	-	-	●, 97.9%
MSHR1132	ST1850	-	-	-	-	-	●, 100%	●, 100%	●, 100%	●, 100%	-	-	●, 97.9%
2588STDY5763869	ST2250	●, 100%	●, 100%	●, 100%	-	-	-	-	-	-	-	-	●, 97.9%
2588STDY5890629	ST2250	●, 100%	●, 100%	●, 100%	●, 100%	-	-	-	-	-	-	-	●, 97.9%
2588STDY5890661	ST2250	●, 100%	●, 100%	●, 100%	●, 100%	-	-	-	-	-	-	-	●, 97.9%
2588STDY5890701	ST2250	●, 100%	●, 96.9%	●, 100%	●, 100%	-	-	-	-	-	-	-	●, 97.9%
2588STDY5890708	ST2250	●, 100%	●, 100%	●, 100%	-	-	-	-	-	-	-	-	●, 97.9%
2588STDY5890732	ST2250	●, 100%	●, 100%	●, 100%	●, 100%	-	-	-	-	-	-	-	●, 97.9%
2588STDY5890733	ST2250	●, 100%	●, 100%	●, 100%	●, 100%	-	-	-	-	-	-	-	●, 97.9%
2588STDY5890798	ST2250	●, 100%	●, 100%	●, 100%	●, 100%	-	-	-	-	-	-	-	●, 97.9%
2588STDY5890811	ST2250	●, 100%	●, 100%	●, 100%	●, 100%	-	-	-	-	-	-	-	●, 97.9%
2588STDY5890910	ST1223	-	-	●, 96.4%	-	●, 95.9%	-	-	●, 96.5%	-	-	-	●, 97.9%
2588STDY5890934	ST2198	-	-	-	●, 98.0%	-	-	-	-	-	-	●, 98.1%	●, 97.9%
2588STDY5962135	ST2250	●, 100%	●, 100%	●, 100%	-	-	-	-	-	-	-	-	●, 97.9%
D7903	ST1223	-	-	-	-	●, 95.9%	-	-	-	-	-	-	●, 97.9%
H1540	ST2250	●, 100%	●, 100%	-	-	-	-	-	-	-	-	-	●, 97.9%
H1604	ST2250	●, 100%	-	-	-	-	-	-	-	-	-	-	●, 97.9%
H1826	ST2250	●, 100%	●, 100%	-	-	-	-	-	-	-	-	-	●, 97.9%
H1864	ST2250	●, 100%	●, 100%	●, 100%	-	-	-	-	-	-	●, 97.4%	-	●, 97.9%
H1955	ST2793	-	-	●, 97.3%	-	-	-	-	-	-	-	-	●, 97.9%
H2179	ST2250	●, 100%	●, 100%	●, 99.4%	-	-	-	-	-	-	-	-	●, 97.9%
M3040	ST2250	●, 100%	●, 100%	●, 100%	●, 100%	-	-	-	-	-	-	-	●, 97.9%
M4143	ST2250	●, 100%	●, 100%	●, 100%	●, 100%	-	-	-	-	-	-	-	●, 97.9%
M4146	ST2250	●, 100%	●, 100%	-	-	-	-	-	-	-	-	-	●, 97.9%
M4148	ST2250	●, 100%	●, 100%	-	-	-	-	-	-	-	-	-	●, 97.9%
M4185	ST1223	-	-	●, 96.4%	-	●, 95.6%	-	-	●, 96.6%	-	-	-	●, 97.9%
M4528	ST1223	-	-	-	-	●, 95.9%	-	-	●, 96.6%	●, 96.3%	-	-	●, 97.9%
M4611	ST2250	●, 100%	●, 100%	●, 100%	●, 100%	-	-	-	-	-	-	-	●, 97.9%
M5200	ST2793	-	-	●, 97.3%	-	-	-	-	-	●, 96.7%	-	-	●, 97.9%
M5219	ST2793	-	-	●, 97.3%	-	-	-	-	-	●, 96.7%	-	-	●, 97.9%
M5224	ST2793	-	-	●, 97.3%	-	-	-	-	-	●, 96.7%	-	-	●, 97.9%
O-1	ST2250	●, 100%	●, 100%	-	-	-	-	-	-	-	-	-	●, 97.9%
O-10	ST2250	●, 100%	●, 100%	-	-	-	-	-	-	-	-	-	●, 97.9%
O-2	unkown	●, 100%	●, 100%	●, 100%	●, 99.4%	-	-	-	-	-	-	-	●, 97.9%
O-3	ST2250	●, 100%	●, 100%	-	-	-	-	-	-	-	-	-	●, 97.9%
O-4	ST2250	●, 100%	●, 100%	●, 100%	●, 100%	-	-	-	-	-	-	-	●, 97.9%
O-5	ST2250	●, 100%	●, 100%	-	-	-	-	-	-	-	-	-	●, 97.9%
O-6	ST2250	●, 100%	●, 100%	●, 100%	●, 100%	-	-	-	-	-	-	-	●, 97.9%
O-9	ST2854	-	-	-	-	-	-	-	-	-	-	-	-
G18754525	ST2250	●, 100%	●, 100%	●, 100%	●, 100%	-	-	-	-	-	-	-	●, 97.9%
G18754612	ST2250	●, 100%	●, 100%	●, 100%	●, 100%	-	-	-	-	-	-	-	●, 97.9%
G18754695	ST2250	●, 100%	●, 100%	●, 100%	●, 100%	-	-	-	-	-	-	-	●, 97.9%
G18754745	ST2250	●, 100%	●, 100%	●, 100%	●, 100%	-	-	-	-	-	-	-	●, 97.9%
G18754754	ST2793	-	-	●, 97.3%	-	-	-	-	-	●, 96.7%	-	-	●, 97.9%
G18760340	ST2250	●, 100%	●, 100%	●, 100%	●, 100%	-	-	-	-	-	-	-	●, 97.9%
G18760797	ST2793	-	-	●, 97.3%	-	-	-	-	-	●, 96.7%	-	-	●, 97.9%
G18761989	ST2793	-	-	●, 97.3%	-	-	-	-	-	●, 96.7%	-	-	●, 97.9%
G18762363	ST2793	-	-	●, 97.3%	-	-	-	-	-	●, 96.7%	-	-	●, 97.9%
G18762364	ST2793	-	-	●, 97.3%	-	-	-	-	-	●, 96.7%	-	-	●, 97.9%
G18762634	ST2793	-	-	●, 97.3%	-	-	-	-	-	●, 96.7%	-	-	●, 97.9%
G18763441	ST2793	-	-	●, 97.3%	-	-	-	-	-	●, 96.7%	-	-	●, 97.9%
MGYG-HGUT-02548	ST2250	●, 100%	●, 99.9%	●, 100%	●, 100%	-	-	-	-	-	-	-	●, 97.9%
RIVM02149_Sar	ST2250	●, 100%	●, 100%	●, 100%	●, 100%	-	-	-	-	-	-	-	●, 97.9%
MUMC_argenteus_1	ST1850	-	-	-	-	-	●, 100%	●, 100%	●, 100%	●, 100%	-	-	●, 97.9%
T151204	ST2250	●, 100%	●, 100%	●, 100%	●, 100%	-	-	-	-	-	-	-	●, 97.9%
1D-1445	ST2250	●, 100%	●, 100%	●, 100%	●, 100%	-	-	-	-	-	-	-	●, 97.9%
1D-1902	ST2250	●, 100%	●, 100%	●, 100%	●, 100%	-	-	-	-	-	-	-	●, 97.9%
1D-304	ST2250	●, 100%	●, 100%	●, 99.9%	●, 100%	-	-	-	-	-	-	-	●, 97.9%
M260_MSHR	ST2198	-	-	-	-	-	-	-	-	-	-	●, 99.9%	●, 98.0%
FJABA32044V6S1	unkown	-	-	-	-	-	-	-	-	●, 96.3%	-	-	●, 97.9%
LBSA043	ST2250	●, 99.9%	●, 100%	●, 100%	●, 100%	-	-	-	-	-	-	-	●, 97.9%
M051_MSHR	ST1223	-	-	-	-	●, 95.9%	-	-	●, 96.5%	●, 96.4%	-	-	●, 97.9%
H115100079	ST2793	-	-	-	-	-	-	-	●, 96.3%	●, 96.8%	-	-	●, 97.9%
NCTC13711	ST1850	-	-	-	-	-	●, 100%	●, 100%	●, 100%	●, 100%	-	-	●, 97.9%
SH3	ST2250	●, 100%	●, 100%	●, 100%	●, 100%	-	-	-	-	-	-	-	●, 97.9%
3688STDY6124917	ST2198	-	-	-	●, 98.0%	-	-	-	-	●, 96.4%	-	●, 98.1%	●, 97.9%
3688STDY6124978	ST2250	●, 100%	●, 100%	●, 100%	●, 100%	-	-	-	-	-	-	-	●, 97.9%
3688STDY6125062	ST2250	●, 100%	●, 100%	●, 100%	●, 99.9%	-	-	-	-	-	-	-	●, 97.9%
3688STDY6125063	ST2250	●, 100%	●, 100%	●, 100%	●, 100%	-	-	-	-	-	-	-	●, 97.9%
3688STDY6125064	ST2250	●, 100%	●, 100%	●, 100%	●, 100%	-	-	-	-	-	-	-	●, 97.9%
3688STDY6125065	ST2250	●, 100%	●, 100%	●, 100%	●, 100%	-	-	-	-	-	-	-	●, 97.9%
3688STDY6125066	ST2250	●, 100%	●, 100%	●, 100%	●, 100%	-	-	-	-	-	-	-	●, 97.9%
3688STDY6125067	ST2250	●, 100%	●, 100%	●, 100%	●, 100%	-	-	-	-	-	-	-	●, 97.9%
3688STDY6125068	ST2250	●, 100%	●, 100%	●, 100%	●, 100%	-	-	-	-	-	-	-	●, 97.9%
3688STDY6125069	ST2250	●, 100%	●, 100%	●, 100%	●, 100%	-	-	-	-	-	-	-	●, 97.9%
3688STDY6125070	ST2250	●, 100%	●, 100%	●, 100%	●, 100%	-	-	-	-	-	-	-	●, 97.9%
3688STDY6125071	ST2250	●, 100%	●, 100%	●, 100%	●, 100%	-	-	-	-	-	-	-	●, 97.9%
3688STDY6125072	ST2250	●, 100%	●, 100%	●, 100%	●, 100%	-	-	-	-	-	-	-	●, 97.9%
3688STDY6125073	ST2250	●, 100%	●, 100%	●, 100%	●, 100%	-	-	-	-	-	-	-	●, 97.9%
3688STDY6125074	ST2250	●, 100%	●, 100%	●, 100%	●, 100%	-	-	-	-	-	-	-	●, 97.9%
3688STDY6125075	ST2250	●, 100%	●, 100%	●, 100%	●, 100%	-	-	-	-	-	-	-	●, 97.9%
3688STDY6125076	ST2250	●, 100%	●, 100%	●, 100%	●, 100%	-	-	-	-	-	-	-	●, 97.9%
3688STDY6125077	ST2250	●, 100%	●, 100%	●, 100%	●, 100%	-	-	-	-	-	-	-	●, 97.9%
3688STDY6125078	ST2250	●, 100%	●, 100%	●, 100%	●, 100%	-	-	-	-	-	-	-	●, 97.9%
3688STDY6125079	ST2250	●, 100%	●, 100%	●, 100%	●, 100%	-	-	-	-	-	-	-	●, 97.9%
3688STDY6125080	ST2250	●, 100%	●, 100%	●, 100%	●, 100%	-	-	-	-	-	-	-	●, 97.9%
3688STDY6125081	ST2250	●, 100%	●, 100%	●, 100%	●, 100%	-	-	-	-	-	-	-	●, 97.9%
3688STDY6125082	ST2250	●, 100%	●, 100%	●, 100%	●, 100%	-	-	-	-	-	-	-	●, 97.9%
3688STDY6125083	ST2250	●, 100%	●, 100%	●, 100%	●, 100%	-	-	-	-	-	-	-	●, 97.9%
3688STDY6125084	ST2250	●, 100%	●, 100%	●, 100%	●, 100%	-	-	-	-	-	-	-	●, 97.9%
3688STDY6125085	ST2250	●, 100%	●, 100%	●, 100%	●, 100%	-	-	-	-	-	-	-	●, 97.9%
3688STDY6125086	ST2250	●, 100%	●, 100%	●, 100%	●, 100%	-	-	-	-	-	-	-	●, 97.9%
3688STDY6125087	ST2250	●, 100%	●, 100%	●, 100%	●, 100%	-	-	-	-	-	-	-	●, 97.9%
3688STDY6125088	ST2250	●, 100%	●, 100%	●, 100%	●, 100%	-	-	-	-	-	-	-	●, 97.9%
3688STDY6125089	ST2250	●, 100%	●, 100%	●, 100%	●, 100%	-	-	-	-	-	-	-	●, 97.9%
3688STDY6125090	ST2250	●, 100%	●, 100%	●, 100%	●, 100%	-	-	-	-	-	-	-	●, 97.9%
3688STDY6125091	ST2250	●, 100%	●, 100%	●, 100%	●, 100%	-	-	-	-	-	-	-	●, 97.9%
3688STDY6125092	unkown	●, 100%	●, 100%	●, 100%	●, 100%	-	-	-	-	-	-	-	●, 97.9%
3688STDY6125093	ST2250	●, 100%	●, 100%	●, 100%	●, 100%	-	-	-	-	-	-	-	●, 97.9%
3688STDY6125100	ST2250	●, 100%	●, 100%	●, 100%	●, 100%	-	-	-	-	-	-	-	●, 97.9%
3688STDY6125105	ST2250	●, 100%	●, 100%	●, 100%	●, 100%	-	-	-	-	-	-	-	●, 97.9%
3688STDY6125106	ST2250	●, 100%	●, 100%	●, 100%	●, 100%	-	-	-	-	-	-	-	●, 97.9%
3688STDY6125108	ST2250	●, 100%	●, 100%	●, 100%	●, 100%	-	-	-	-	-	-	-	●, 97.9%
3688STDY6125109	ST2250	●, 100%	●, 100%	●, 100%	●, 100%	-	-	-	-	-	-	-	●, 97.9%
3688STDY6125110	ST2250	●, 100%	●, 100%	●, 100%	●, 100%	-	-	-	-	-	-	-	●, 97.9%
3688STDY6125111	ST2250	●, 100%	●, 100%	●, 100%	●, 100%	-	-	-	-	-	-	-	●, 97.9%
3688STDY6125112	ST2250	●, 100%	●, 100%	●, 100%	●, 100%	-	-	-	-	-	-	-	●, 97.9%
3688STDY6125113	ST2250	●, 100%	●, 100%	●, 100%	●, 100%	-	-	-	-	-	-	-	●, 97.9%
3688STDY6125114	ST2250	●, 100%	●, 100%	●, 100%	●, 100%	-	-	-	-	-	-	-	●, 97.9%
3688STDY6125115	ST2250	●, 100%	●, 100%	●, 100%	●, 100%	-	-	-	-	-	-	-	●, 97.9%
3688STDY6125116	ST2250	●, 100%	●, 100%	●, 100%	●, 100%	-	-	-	-	-	-	-	●, 97.9%
3688STDY6125117	ST2250	●, 100%	●, 100%	●, 100%	●, 100%	-	-	-	-	-	-	-	●, 97.9%
3688STDY6125118	ST2250	●, 100%	●, 100%	●, 100%	●, 100%	-	-	-	-	-	-	-	●, 97.9%
3688STDY6125119	ST2250	●, 100%	●, 100%	●, 100%	●, 100%	-	-	-	-	-	-	-	●, 97.9%
3688STDY6125120	ST2250	●, 100%	●, 100%	●, 100%	●, 100%	-	-	-	-	-	-	-	●, 97.9%
3688STDY6125121	ST2250	●, 100%	●, 100%	●, 100%	●, 100%	-	-	-	-	-	-	-	●, 97.9%
3688STDY6125122	ST2250	●, 100%	●, 100%	●, 100%	●, 100%	-	-	-	-	-	-	-	●, 97.9%
3688STDY6125123	ST2250	●, 100%	●, 100%	●, 100%	●, 100%	-	-	-	-	-	-	-	●, 97.9%
3688STDY6125124	ST2250	●, 100%	●, 100%	●, 100%	●, 100%	-	-	-	-	-	-	-	●, 97.9%
3688STDY6125125	ST2250	●, 100%	●, 100%	●, 100%	●, 100%	-	-	-	-	-	-	-	●, 97.9%
3688STDY6125126	ST2250	●, 100%	●, 100%	●, 100%	●, 100%	-	-	-	-	-	-	-	●, 97.9%
3688STDY6125127	ST2198	-	-	-	●, 98.0%	-	-	-	-	●, 98.0%	-	●, 99.9%	●, 98.1%
3688STDY6125128	ST1223	-	-	●, 96.0%	-	●, 95.9%	-	-	●, 96.4%	-	-	-	●, 97.9%
3688STDY6125129	ST2854	-	-	●, 96.0%	-	-	-	-	-	-	-	-	-
3688STDY6125130	ST2854	-	-	-	-	-	-	-	-	-	-	-	-
3688STDY6125131	ST2250	●, 100%	●, 100%	●, 100%	●, 100%	-	-	-	-	-	-	-	●, 97.9%
3688STDY6125132	ST1223	-	-	●, 96.3%	-	●, 95.9%	-	-	●, 96.6%	-	-	-	●, 97.9%
3688STDY6125133	ST1223	-	-	●, 96.3%	-	●, 95.9%	-	-	●, 96.6%	-	-	-	●, 97.9%
3688STDY6125134	ST2854	-	-	●, 96.0%	-	-	-	-	-	-	-	-	-
3688STDY6125135	ST1223	-	-	●, 96.4%	-	●, 95.9%	-	-	●, 96.6%	-	-	-	●, 97.9%
3688STDY6125136	ST2250	●, 100%	●, 100%	●, 100%	●, 100%	-	-	-	-	-	-	-	●, 97.9%
3688STDY6125137	ST2198	-	-	-	●, 98.1%	-	-	-	-	●, 96.4%	-	●, 99.9%	●, 98.1%
3688STDY6125138	ST2250	●, 100%	●, 100%	●, 100%	●, 100%	-	-	-	-	-	-	-	●, 97.9%
3688STDY6125139	ST1223	-	-	●, 96.3%	-	●, 95.9%	-	-	●, 96.6%	-	-	-	●, 97.9%
3688STDY6125140	ST2250	●, 100%	●, 100%	●, 100%	●, 100%	-	-	-	-	-	-	-	●, 97.9%
3688STDY6125143	ST1223	-	-	●, 96.4%	-	●, 95.9%	-	-	●, 96.6%	-	-	-	●, 97.9%
PR02	ST2854	-	-	-	-	-	-	-	-	-	-	-	-
F87619	ST2250	-	●, 99.9%	●, 100%	-	-	-	-	-	-	-	-	●, 97.9%
M21126	ST2250	●, 100%	●, 100%	●, 100%	●, 100%	-	-	-	-	-	-	-	●, 97.9%
1299_SAUR	ST2250	●, 100%	●, 100%	●, 99.6%	-	-	-	-	-	-	-	-	●, 97.9%
SCPM-O-B-8378	ST2250	●, 100%	●, 100%	●, 100%	●, 100%	-	-	-	-	-	-	-	●, 97.9%
ABFQM	ST2198	-	-	-	●, 98.0%	-	-	-	-	●, 96.4%	-	●, 99.9%	●, 98.1%
CCUG69384	ST2250	●, 100%	●, 100%	●, 99.9%	●, 100%	-	-	-	-	-	-	-	●, 97.9%
CCUG69385	ST1223	-	-	●, 96.3%	-	●, 95.9%	-	-	●, 96.6%	-	-	-	●, 97.9%
RK308	ST2250	●, 100%	●, 100%	●, 100%	●, 100%	-	-	-	-	-	-	-	●, 97.9%
SJTU-F20124	ST3261	-	-	-	-	●, 95.7%	-	-	-	-	-	-	●, 98.0%

SJTU-F20419	ST2250	●, 100%	●, 100%	●, 100%	●, 100%	-	-	-	-	-	-	-	●, 97.9%
SJTU-F21164	ST2250	●, 100%	●, 100%	●, 100%	●, 100%	-	-	-	-	-	-	-	●, 97.9%
SJTU-F21224	ST2250	●, 100%	●, 99.9%	●, 100%	●, 100%	-	-	-	-	-	-	-	●, 97.9%
SJTU-F21285	ST2250	●, 100%	●, 99.8%	●, 100%	●, 100%	-	-	-	-	-	-	-	●, 97.9%
BN75	ST2198	-	-	-	●, 98.1%	-	-	-	-	●, 96.4%	-	●, 99.9%	●, 98.1%
DSM28299	ST1850	-	-	-	-	-	-	-	●, 100%	●, 100%	-	-	●, 97.9%
XNO62	ST2250	●, 100%	●, 100%	●, 100%	●, 100%	-	-	-	-	-	-	-	●, 97.9%
XNO106	ST2250	●, 100%	●, 100%	●, 100%	●, 100%	-	-	-	-	-	-	-	●, 97.9%
PH3432	ST2250	●, 100%	●, 100%	●, 100%	●, 100%	-	-	-	-	-	-	-	●, 97.9%
ST2250	ST2250	●, 100%	●, 100%	●, 100%	●, 100%	-	-	-	-	-	-	-	●, 97.9%
PHL3431	ST2250	●, 100%	●, 100%	●, 100%	●, 100%	-	-	-	-	-	-	-	●, 97.9%
PHL3433	ST2250	●, 100%	●, 100%	●, 100%	●, 100%	-	-	-	-	-	-	-	●, 97.9%
Arg23	unkown	-	-	-	-	-	-	-	●, 96.4%	●, 97.7%	-	-	●, 97.9%
ArgPVL	ST2250	●, 100%	●, 100%	●, 100%	●, 100%	-	-	-	-	-	-	-	●, 97.9%
B3-25B	ST2198	-	-	-	●, 98.1%	-	-	-	-	●, 96.4%	-	●, 99.9%	●, 98.1%
5506	ST2250	●, 100%	●, 100%	-	●, 99.9%	-	-	-	-	-	-	-	●, 97.9%
18A183	ST1223	-	-	●, 96.4%	-	●, 95.9%	-	-	●, 96.5%	-	-	-	●, 97.9%
CFSA15SA062	ST2250	●, 100%	●, 99.9%	●, 100%	●, 100%	-	-	-	-	-	-	-	●, 97.9%
CFSA17SA018	ST1223	-	-	●, 96.4%	-	●, 95.9%	-	-	●, 96.6%	-	-	-	●, 97.9%
CFSA17SA053	ST1223	-	-	●, 96.4%	-	●, 95.9%	-	-	●, 96.6%	-	-	-	●, 97.9%
CFSA17SA054	ST1223	-	-	●, 96.4%	-	●, 95.9%	-	-	●, 96.6%	-	-	-	●, 97.9%
058S_SR	ST2250	●, 100%	●, 100%	●, 99.9%	-	-	-	-	-	-	-	-	●, 97.9%
117N_SR	ST2250	●, 100%	●, 100%	●, 100%	●, 100%	-	-	-	-	-	-	-	●, 97.9%
305N_SR	unkown	●, 100%	●, 100%	●, 100%	-	-	-	-	-	-	-	-	●, 97.9%
752S_SR	ST2250	●, 99.9%	●, 100%	●, 99.8%	●, 100%	-	-	-	-	-	-	-	●, 97.9%
BAL1928	ST2250	●, 100%	●, 100%	●, 100%	●, 100%	-	-	-	-	-	-	-	●, 97.9%
BAL1935	ST2250	●, 100%	●, 100%	●, 100%	●, 100%	-	-	-	-	-	-	-	●, 97.9%
BAL1945	ST2250	●, 100%	●, 100%	●, 100%	●, 100%	-	-	-	-	-	-	-	●, 97.9%
BAL2160	ST1223	-	-	●, 96.3%	-	●, 95.9%	-	-	●, 96.6%	-	-	-	●, 97.9%
BC15-02467	ST2250	●, 100%	●, 100%	●, 100%	●, 100%	-	-	-	-	-	-	-	●, 97.9%
NS2714	ST2250	●, 100%	●, 99.9%	●, 100%	●, 100%	-	-	-	-	-	-	-	●, 97.9%
NS3791	ST1223	-	-	-	-	●, 95.9%	-	-	●, 96.6%	-	-	-	●, 97.9%
NS425	ST2250	●, 100%	●, 99.9%	●, 100%	●, 100%	-	-	-	-	-	-	-	●, 97.9%
DSM28299	ST1850	-	-	-	-	-	-	-	●, 100%	●, 100%	-	-	●, 97.9%
MC2	ST2250	●, 100%	●, 100%	●, 100%	●, 99.9%	-	-	-	-	-	-	-	●, 97.9%
MC3	ST2250	●, 100%	●, 100%	●, 100%	●, 99.9%	-	-	-	-	-	-	-	●, 97.9%
MC4	ST2250	●, 100%	●, 100%	●, 100%	●, 99.9%	-	-	-	-	-	-	-	●, 97.9%
PHL1144	ST1223	-	-	●, 96.4%	-	●, 95.9%	-	-	-	-	-	-	●, 97.9%
PHL2411	ST2198	-	-	-	-	-	-	-	-	-	-	●, 99.9%	●, 98.0%
PHL2420	ST2250	-	-	-	-	-	-	-	-	-	-	-	●, 97.9%
PHL3446	ST2250	●, 100%	●, 100%	-	-	-	-	-	-	-	-	-	●, 97.9%
PHL4226	ST2250	-	-	-	-	-	-	-	-	-	-	-	●, 97.9%
PHL4313	ST1223	-	-	●, 96.4%	-	●, 95.9%	-	-	●, 96.6%	-	-	-	●, 97.9%
PHL4553	unkown	-	-	-	-	-	-	-	-	-	-	●, 99.9%	●, 98.0%
PHL4815	ST2250	-	-	-	-	-	-	-	-	-	-	-	●, 97.9%
PHL5740	ST1223	-	-	●, 96.4%	-	●, 95.9%	-	-	●, 96.6%	-	-	-	●, 97.9%
PHL6318	ST2250	●, 100%	●, 99.1%	-	-	-	-	-	-	-	-	-	●, 97.9%
PHL6344	ST2250	●, 100%	●, 100%	-	-	-	-	-	-	-	-	-	●, 97.9%
PHL8605	ST2250	-	-	-	-	-	-	-	-	-	-	-	●, 97.8%
PHL8642	unkown	-	-	-	-	-	-	-	-	-	-	-	●, 97.9%
WU1	ST2250	●, 99.9%	●, 100%	●, 100%	●, 100%	-	-	-	-	-	-	-	●, 97.9%
WU2	ST2250	●, 100%	●, 100%	●, 100%	●, 99.9%	-	-	-	-	-	-	-	●, 97.9%
WU3	ST2250	●, 100%	●, 100%	●, 100%	●, 100%	-	-	-	-	-	-	-	●, 97.9%
JARB-RP-0005 (SG-01)	ST2250	●, 100%	●, 100%	-	-	-	-	-	-	-	-	-	●, 97.9%
JARB-RP-0006 (SG-02)	ST2198	-	-	-	●, 98.1%	-	-	-	-	●, 96.4%	-	●, 99.9%	●, 98.0%
JARB-RP-0007 (SG-03)	ST1223	-	-	●, 96.2%	-	●, 95.9%	-	-	●, 96.6%	-	-	-	●, 97.9%
JARB-RP-0008 (SG-04)	ST2250	●, 100%	●, 100%	●, 100%	●, 100%	-	-	-	-	-	-	-	●, 97.9%
JARB-RP-0009 (SG-05-1)	ST2250	●, 100%	●, 100%	●, 100%	●, 100%	-	-	-	-	-	-	-	●, 97.9%
JARB-RP-0010 (SG-05-2)	ST2250	●, 100%	●, 100%	●, 100%	●, 100%	-	-	-	-	-	-	-	●, 97.9%
JARB-RP-0011 (SG-06)	ST1223	-	-	-	-	●, 95.9%	-	-	-	-	-	-	●, 97.9%
JARB-RP-0012 (SG-07)	ST1223	-	-	-	-	●, 95.9%	-	-	●, 96.6%	-	-	-	●, 97.9%
JARB-RP-0013 (SG-08)	ST2198	-	-	-	●, 98.1%	-	-	-	-	●, 96.4%	-	●, 99.9%	●, 98.0%
JARB-RP-0014 (SG-09)	ST2250	●, 99.9%	●, 100%	●, 100%	●, 100%	-	-	-	-	-	-	-	●, 97.9%
JARB-RP-0015 (SG-10)	ST2198	-	-	-	●, 98.1%	-	-	-	-	●, 96.4%	-	●, 99.9%	●, 98.0%
JARB-RP-0016 (SG-11)	ST2250	●, 100%	●, 100%	●, 100%	●, 100%	-	-	-	-	-	-	-	●, 97.9%
JARB-RP-0019 (SG-14)	ST2198	-	-	-	●, 98.1%	-	-	-	-	●, 96.4%	-	●, 99.9%	●, 98.0%
JARB-RP-0021 (SG-16)	ST2250	●, 100%	●, 100%	●, 100%	●, 100%	-	-	-	-	-	-	-	●, 97.9%
JARB-RP-0022 (SG-17)	ST1223	-	-	-	-	●, 95.9%	-	-	●, 96.6%	-	-	-	●, 97.9%
JARB-RP-0023 (SG-18)	ST1223	-	-	●, 96.2%	-	●, 95.9%	-	-	●, 96.6%	-	-	-	●, 97.9%
JARB-RP-0025 (SG-20)	ST2198	-	-	-	●, 98.0%	-	-	-	-	●, 96.4%	-	●, 99.9%	●, 98.0%
JARB-RP-0026 (SG-21)	ST2250	●, 100%	●, 100%	●, 100%	●, 100%	-	-	-	-	-	-	-	●, 97.9%
JARB-RP-0027 (SG-22)	ST2250	●, 100%	●, 100%	●, 100%	●, 100%	-	-	-	-	-	-	-	●, 97.9%
JARB-RP-0028 (SG-23)	ST2250	●, 100%	●, 100%	●, 100%	-	-	-	-	-	-	-	-	●, 97.9%
JARB-RP-0029 (SG-25)	ST2250	●, 100%	●, 100%	●, 100%	●, 100%	-	-	-	-	-	-	-	●, 97.9%
JH5333	ST2250	●, 100%	●, 100%	●, 100%	●, 100%	-	-	-	-	-	-	-	●, 97.9%
JH5336	ST1223	-	-	●, 96.3%	-	●, 95.9%	-	-	●, 96.6%	-	-	-	●, 97.9%
JH5337	ST1223	-	-	●, 96.3%	-	●, 95.9%	-	-	●, 96.6%	-	-	-	●, 97.9%
JH5338	ST2250	●, 100%	●, 100%	●, 100%	●, 100%	-	-	-	-	-	-	-	●, 97.9%
JH5340	ST1223	-	-	●, 96.3%	-	●, 95.9%	-	-	●, 96.6%	-	-	-	●, 97.9%
JH6199	ST2250	●, 100%	●, 100%	-	●, 100%	-	-	-	-	-	-	-	●, 97.9%
JH6211	ST2250	●, 100%	●, 100%	-	-	-	-	-	-	-	-	-	●, 97.9%
P217	ST1223	-	-	-	-	●, 95.9%	-	-	-	-	-	-	●, 97.9%
P309	ST2854	-	-	-	-	-	-	-	-	-	-	-	-
P476	ST1223	-	-	●, 96.4%	-	●, 95.9%	-	-	●, 96.6%	-	-	-	●, 97.9%
P527	ST1223	-	-	●, 96.4%	-	●, 95.9%	-	-	●, 96.6%	-	-	-	●, 97.9%
P571	ST2854	-	-	●, 96.3%	-	-	-	-	-	-	-	-	-
P729	ST1223	-	-	●, 96.4%	-	●, 95.9%	-	-	●, 96.6%	-	-	-	●, 97.9%
SARG1271	ST2250	●, 100%	●, 100%	●, 100%	●, 100%	-	-	-	-	-	-	-	●, 97.9%
SARG1913	ST2250	●, 100%	●, 100%	●, 100%	●, 100%	-	-	-	-	-	-	-	●, 97.9%
SARG2343	ST2250	●, 100%	●, 100%	-	-	-	-	-	-	-	-	-	●, 97.9%
SARG3091	ST2250	●, 100%	●, 100%	●, 100%	●, 100%	-	-	-	-	-	-	-	●, 97.9%
SARG5253	unkown	●, 100%	●, 100%	●, 100%	●, 100%	-	-	-	-	-	-	-	●, 97.9%
